# Rise of post-pandemic resilience across the distrust ecosystem

**DOI:** 10.1038/s41598-023-42893-6

**Published:** 2023-09-20

**Authors:** Lucia Illari, Nicholas J. Restrepo, Neil F. Johnson

**Affiliations:** 1 Dynamic Online Networks Laboratory, George Washington University, Washington, DC 20052 USA; 2 Physics Department, George Washington University, Washington, DC 20052 USA; 3 ClustrX LLC, Washington, DC USA

**Keywords:** Health policy, Quality of life, Nonlinear phenomena

## Abstract

Why does online distrust (e.g., of medical expertise) continue to grow despite numerous mitigation efforts? We analyzed changing discourse within a Facebook ecosystem of approximately 100 million users who were focused pre-pandemic on vaccine (dis)trust. Post-pandemic, their discourse interconnected multiple non-vaccine topics and geographic scales within and across communities. This interconnection confers a unique, system-level (i.e., at the scale of the full network) resistance to mitigations targeting isolated topics or geographic scales—an approach many schemes take due to constrained funding. For example, focusing on local health issues but not national elections. Backed by numerical simulations, we propose counterintuitive solutions for more effective, scalable mitigation: utilize “glocal” messaging by blending (1) strategic topic combinations (e.g., messaging about specific diseases with climate change) and (2) geographic scales (e.g., combining local and national focuses).

## Introduction

Distrust and its associated mis/disinformation—however defined—is now a widespread threat to public health (e.g., abortion, COVID-19, mpox (previously called monkeypox)), science (e.g., climate change), election processes and even national security^1–5^. The pandemic exacerbated this issue as many people turned to their trusted online communities for advice and to share distrust of official health messaging^6–18^. Within a month of the U.S. national emergency declaration^19^, Facebook—the largest and most widely used social media platform—saw a 50% increase in messaging and 70% increase in time spent^20^, driving its monthly active users to 2.6 billion.

To combat the growth of online distrust and mis/disinformation, myriad ingenious mitigation strategies have been introduced and implemented on social media platforms^12,21–38^. For instance, Facebook has funded internal efforts like adding misinformation labels to posts^25^, while The Mercury Project, spearheaded by the Social Science Research Council with support from philanthropic organizations like The Rockefeller Foundation, Robert Wood Johnson Foundation, and Craig Newmark Philanthropies, has funded vaccine promotion campaigns^28^. Depending on a mitigation scheme's funding source, its focus is typically on a specific topic (e.g., COVID-19, elections, climate change), and geographical scale, such as a specific state, national (e.g., APS), or worldwide (e.g., E.U.). However, despite the diversity of mitigation schemes, distrust continues to be widespread.

**Figure 1:**
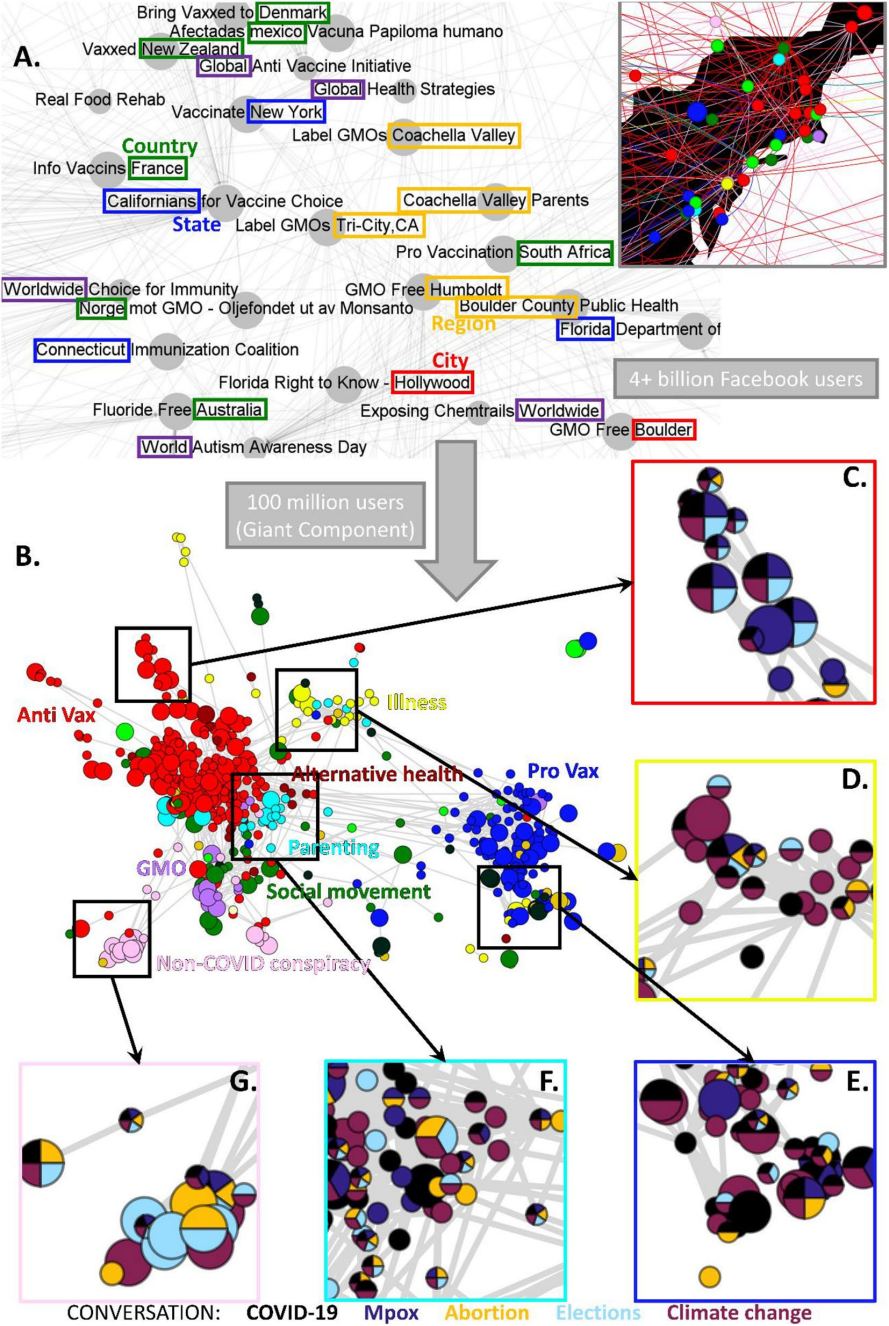
Post-pandemic distrust entangles topics, locations, and geographical scales. (**A**) Illustrative sample of our data^40^. Each circle is a Facebook community (page). Communities promote page-level links to each other. Inset illustrates communities' locations in U.S. north-east. (**B**) Giant connected component of communities classified according to their stance on vaccines. Neutral communities (i.e., non-blue, non-red) are subclassified by their primary interest, e.g., parenting (light blue). Node size indicates geographic scale: large nodes are local communities; small nodes are global ones. (**C**–**G**) Each community's discourse sub-classified by proportion of dominant topics (black is Covid-19, dark purple is mpox, gold is abortion, light blue is elections, dark magenta is climate change). SI Sect. 6 gives full information and shows the complete network.

**Figure 2:**
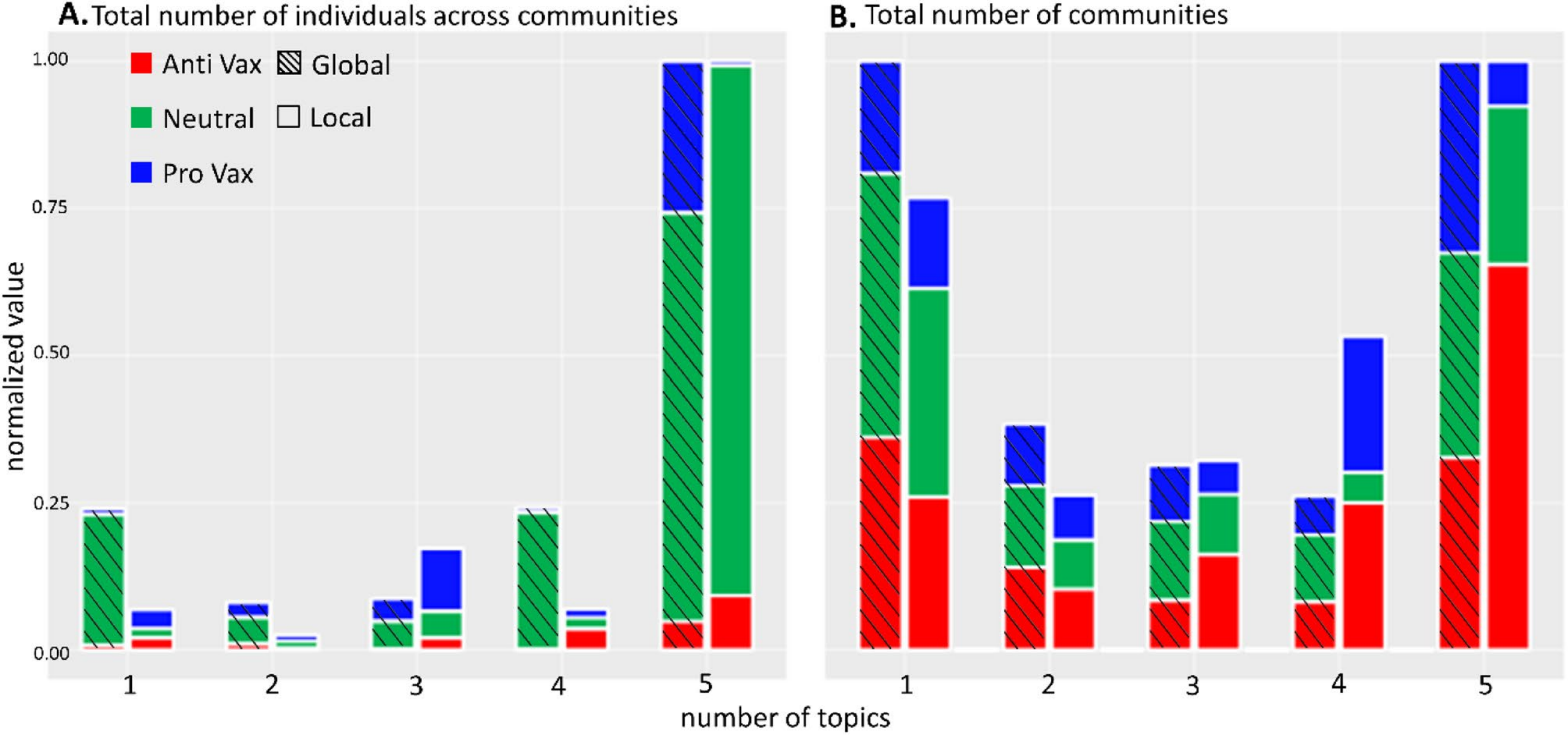
The numbers of topics discussed by (**A**) individuals, and (**B**) communities. Categorized by their geographic scale and stance in the vaccine debate. Global communities are shaded. Results normalized by the maximum value. SI Sect. 9 confirms that these patterns do not arise by chance.

**Figure 3:**
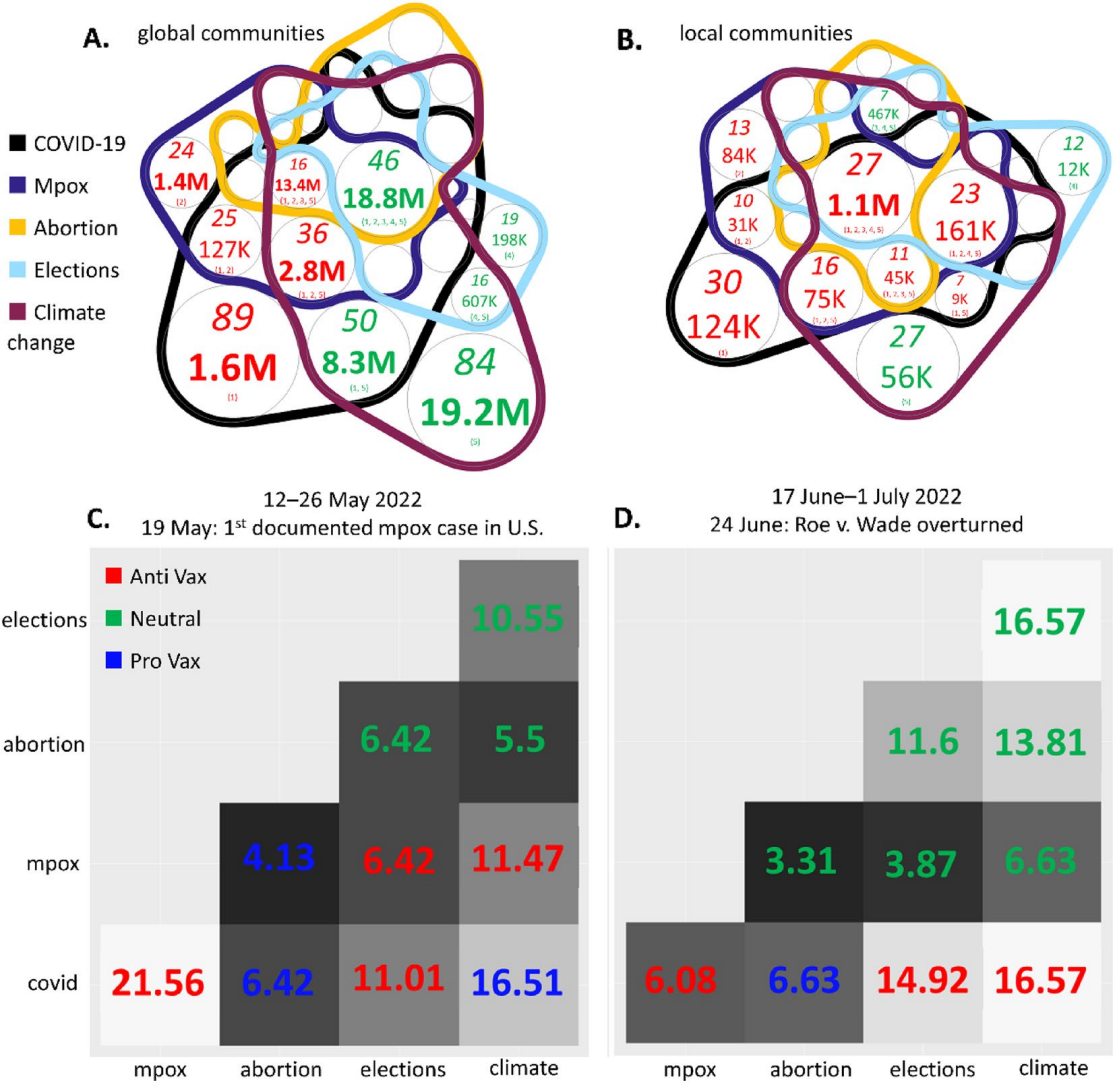
Topic mix within (**A**) global communities, and (**B**) local communities, shown using n-Venn diagrams (see SI Sect. 8 for simple example explaining an n-Venn diagram^61^). Number of communities in italics at top. Number of individuals in middle (in bold if > 1 M). Specific combination of topics at bottom. Number is shown in red (or green) if there is a prevalence of anti-vaccination (or neutral) communities. Only regions with > 3% of total communities are labeled. (**C**,**D**) Heatmaps showing the percentage of communities that have posted about both topics for (**C**) 12 May 2022 to 26 May 2022 around first documented case of mpox in U.S. (19 May); (**D**) 17 June 2022 to 01 July 2022 around 24 June overturning of Roe v. Wade. Black-to-white scale represents low-to-high percentages of communities discussing both topics. Text color indicates vaccine stance of the majority of communities that discuss the two topics. Symmetric squares are left empty.

**Figure 4:**
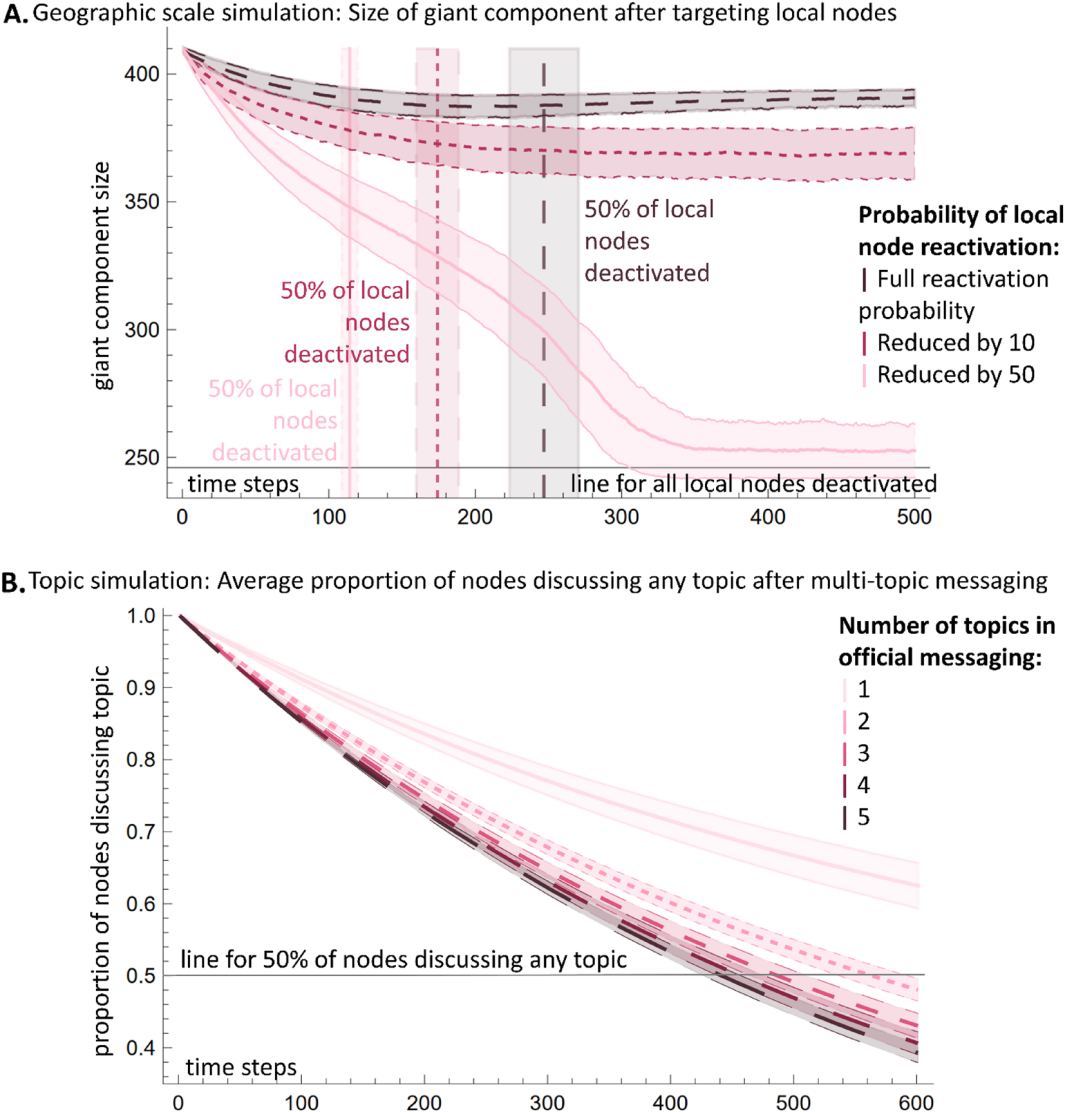
Agent-based simulation results for mitigation schemes. (**A**) Demonstrates the global–local cohesion of the system by plotting the size of the giant component against time. Vertical lines represent $\mu \pm \sigma$ at the point when 50% of local nodes are deactivated. The curves labeled "Full reactivation probability" and "Reduced by 10" stabilize over time, while the "Reduced by 50" curve shows an initial decline before stabilizing slightly above the line that indicates the giant component size when all local nodes are deactivated. (**B**) Single vs multi-topic debunking: proportion of nodes discussing *n* topics versus time. Results show much higher impact for multi-topic messaging.

Here we provide an answer to this question, and the counterintuitive solution that this answer suggests. Specifically, we show that post-pandemic distrust has developed a massive glocal web that—within individual communities and across interconnected communities—blends distinct topics, locations, and geographic scales. This makes it resilient to current mitigation schemes that only focus on a specific topic or geographic scale (e.g. due to funding mandate)^39^. Given that such schemes also operate independently, this suggests widespread distrust would remain resilient even if these schemes were implemented at mass scale. We show this in Figs. 1, 2 and 3 by analyzing the post-pandemic discourse across the Facebook ecosystem of approximately 100 million individuals that—pre-pandemic—was centered on vaccine distrust^40^. Combining this with an agent-based simulation, Fig. 4 shows how this web-of-distrust can be dismantled by making individual mitigation schemes blend topics and scales.

## Methods

To examine how the distrust discourse changed post-pandemic, we revisited the 2019 Facebook ecosystem from Ref.^40^ that had centered around vaccines and comprised interlinked anti-, pro-, and neutral-vaccination Facebook pages. Our full methodology is given in the SI and follows Ref.^40^. Each Facebook page is a community (i.e., node in Fig. 1) with a unique ID, and has nothing to do with community detection in networks. These communities provide spaces where users gather around shared interests, thereby promoting trust among them^41–46^ and potential collective distrust of other issues^6,22,40,47–54^.

Our trained researchers manually and independently classify each community involved in the vaccine debate, with subsequent consensus checks performed in cases of disagreement. This yielded a network of 1356 interlinked communities across countries and languages, with 86.7 million individuals in the largest network component; 211 pro-vaccination communities (blue nodes, Fig. 1B) with 13.0 million individuals; 501 anti-vaccination communities (red nodes, Fig. 1B) with 7.5 million individuals; 644 neutral communities (non-blue or red nodes, Fig. 1B) with 66.2 million individuals. These neutral communities were further sub-categorized by type based on their title and description (e.g., parenting).

The discourse within each of the 1356 communities was categorized by topic prevalence. Though topics outside the five dominant ones (COVID-19, mpox, abortion, climate change, and elections) are mentioned, their frequency is generally much lower (e.g., sports). To categorize the discourse topics within each community, we developed word filters for five non-vaccine topics: COVID-19, mpox, abortion, elections, and climate change. By 'non-vaccine' we mean topics not centered on vaccines in a broad sense; although the COVID-19 and mpox filters included some vaccination-related terms (for e.g., “monkeepox vax’n”), the intent was to capture discourse specifically about these diseases, not vaccines generally. After all, if we had been filtering for general vaccine discussion rather than disease-specific discourse, it would have been difficult to reliably distinguish COVID-19-related posts from mpox-related posts in an automated manner without additional human classification. The filters combined regular expressions and keyword searches across post content, descriptions, image tags, and link text in multiple languages (see SI Sect. 7 for details on the filtering methodology).

A link is shown between two communities (Facebook pages) *A* and *B* when community *A* recommends community *B* to its members at the page level. This creates a prominent hyperlink from *A* to *B* indicating community *A*'s interest in *B*, which is different than if a member of *A* had simply mentioned some content from *B*. A link does not necessarily mean the two communities agree. Instead, it directs the attention of *A*'s members to *B*, and vice versa it exposes *A* to feedback and content from *B*. While not all members will necessarily pay attention, a committed minority of 25% can be enough to influence the stance of an online community^55^.

Of the 1356 communities, 342 identify as local and have around 3.1 million individuals, while the remaining 1014 communities are global with around 83.7 million individuals. In terms of geography, a global community is a page with broad, worldwide focus that is not tied to a specific location, while a local community is focused on a specific geographic area, such as a neighborhood, city, county, state, or country (e.g., "Vaccine information for parents" or "Global Trends" vs "Vaccine information for Los Angeles County parents"). In terms of topic, a global community discusses diverse issues broadly, whereas a local community has a narrow topical focus (e.g., pages discussing only elections). The size of each community can be estimated by the number of likes, given that the average user only likes one Facebook page on average^7^—however, our analysis and findings are not dependent on this.

Thus, the terms ‘global’ and ‘local’ are applied in two dimensions—geographic and topic—to highlight the interconnection between geographic and topic glocality. This dual usage of ‘global’ and ‘local’ elucidates how geographic and topic glocality may interrelate and allow communities to occupy different glocal positions. For instance, a community focused on a narrow topic within a small locality embodies hyperlocality on both dimensions, while one that discusses many topics worldwide embodies hyperglobality. Our analysis aims to demonstrate that post-pandemic, vaccine skepticism discourse expanded beyond just hyperlocal geography and topics to encompass more hyperglobal geography and topics. The terms ‘global’ and ‘local’ effectively convey this evolution across both dimensions.

In our numerical simulations, ‘mitigation’ refers to efforts that aim to counter mis/disinformation, such as fact-checking, verification, public awareness campaigns, collaborative initiatives, research, and global programs, rather than banning communities. These strategies have been implemented to address false narratives related to topics like COVID-19, public health, elections, and climate change^12,21–38^. For instance, Facebook has funded internal efforts like adding misinformation labels to posts, while the Mercury Project has conducted vaccine promotion campaigns^25,28^. Many strategies operate at a national level, like the UK's ‘Don't Feed the Beast’ campaign^23^ while others have a global focus, like the United Nations' VERIFIED initiative^26^. The strategies aim to limit the spread and impact of misinformation by encouraging critical thinking, accurate reporting, and authoritative voices. However, persistent exposure to narratives via social connections can enable reactivation of silenced misinformation.

Thus, ‘deactivation’ refers to temporarily 'silencing' communities by removing their network links as part of a mitigation campaign focused on a particular topic or locality. However, 'reactivation' allows deactivated communities to resume discussing the targeted topic based on continued exposure to narratives via their remaining network connections. This represents the limited, temporary effectiveness of real-world debunking efforts, with misinformation narratives persisting over time. Our agent-based model thus simulates the effects of different mitigation strategies by ‘deactivating’ communities through removing their network connections, then allowing ‘reactivation’ based on remaining links. The geography-focused simulation tests the impact of messaging targeted at either local or global communities, whereas the topic-focused simulation compares single versus multi-topic debunking (see SI Sect. 10 and 11 for full details). The goal is to capture general principles around network resilience despite the known limited impacts of current mitigation strategies.

## Results

Figure 1 shows this web-of-distrust during the period 5/1/2022 to 10/17/2022, which included several significant events: (1) the first confirmed U.S. case of the mpox outbreak^56^; (2) the U.S. Supreme Court's reversal of Roe v. Wade^57^; (3) President Biden signing into law the Inflation Reduction Act^58^; (4) primary and run-off elections ahead of the November midterms^59^. Communities that share more links appear visually closer together and have a higher likelihood of exerting influence on each other through shared content and infiltration. This is because the layout results from a color-agnostic physical calculation (ForceAtlas2) in which nodes repel each other with a force that decays with separation, and linked nodes have an additional attractive spring force^60^.

This web-of-distrust entangles 5 dominant topics within and across communities: abortion, mpox, COVID-19, climate change, elections (panels C–G). This means that within a given community, distrust in one topic can immediately be reinforced by distrust of another topic(s) as well as by the collective distrust of other communities to which it is linked. Hence post-pandemic intervention on one topic and scale invites pushback from distrust on other topics and across scales, making the distrust ecosystem resilient to mitigation schemes that focus on a particular topic and geographic scale. For example, distrust of state elections within a community associated with a small U.S. city is reinforced by distrust in the U.S. federal government on mpox, which is then reinforced by distrust over climate change from another community representing itself as the U.K. mainstream. This global-local entanglement across topics and scales adds new resilience to the distrust ecosystem.

Figure 1 has counterintuitive implications for messaging the public and hence intervening against distrust (see SI for details and statistical analyses). One might expect discussions surrounding elections and abortion to focus on a specific geographical scale within the U.S. due to specific laws, politics, and healthcare systems. However, a chi-square test shows the opposite is true. Among communities only discussing a single topic, 25.21%, 35.14%, 17.65%, 38.71%, and 24.32% that discuss COVID-19, mpox, abortion, elections, and climate change, respectively, are local—in contrast to the expected 31.9% if there were no correlation. This expected value of 31.9% comes from the chi-squared test results, specifically the expected counts under the null hypothesis of no relationship between topic and geographic locale (see SI Sect. 7). This implies that any single-topic messaging around COVID-19, or abortion, or climate change, should have more of a global perspective, while that around mpox and elections should be more local. This difference between mpox and COVID-19 also warns against one-size-fits-all for public health messaging, despite both being emerging diseases.

Moreover, traditional anti-vax communities—a third of which are local—have a lower interest in abortion (Fig. 1C). Climate change is the most popular topic among illness communities, of which only a few are local (Fig. 1D). Parenting communities are closely associated with alternative health communities and discuss many topics (Fig. 1F). Conspiracy theory communities show high interest in abortion and elections and are 50% local (Fig. 1G). GMO communities have the highest percentage of local members (53.8%) as compared to all communities in Fig. 1B for which only 25.2% are local.

Figure 2 confirms the wide distribution across topics and geographic scales of the distrust discourse. For anti-vaccination communities, this is the number of topics about which distrust is being actively promoted. One might expect that as a community addresses more topics, the number of potential flashpoints for internal disagreements would increase—hence there would be less communities and individuals engaging in higher numbers of topics—but the opposite happens. Furthermore, local communities are overrepresented in topics compared to the full dataset: hence future mitigation schemes, including global ones, should be designed such that local communities and their interests feature in a prominent way.

Figure 3A,B show the importance of specific combinations of topics for effective public messaging. The subset of communities discussing all five topics is the third largest but has the highest number of individuals at 19.9 million. There are fewer communities that only discuss mpox, abortion, or elections. There is significant conversation overlap between COVID-19 and climate change, particularly in global communities. Despite the involvement of pro-vaccination communities in these discussions, the dialog is mostly led by communities that do not promote guidance consistent with current scientific consensus—and in many cases, these are communities that actively oppose it, especially at the local level.

The heatmaps in Fig. 3C,D compare how different pairs of topics were discussed across the distrust ecosystem during key 2022 periods. In Fig. 3C, which includes the first U.S. mpox case, we observe a greater proportion of anti-vaccination communities discussing mpox in connection with COVID-19 compared to pro-vaccination communities; the latter continued to focus more on COVID-19 and climate change interactions (see SI Sect. 9 for full details). This distribution suggests potential gaps in authoritative medical guidance about mpox during this early stage. In Fig. 3D, coinciding with the reversal of Roe v. Wade, pro-vaccination communities discussed abortion predominantly in the context of COVID-19, while neutral communities focused on abortion and climate change connections. However, a higher proportion of anti-communities maintained messaging linking COVID-19 with climate and elections. This observed messaging pattern combining dominant topics resembles a phenomenon akin to real multi-virus interference^62^: the strategic blending of topics helps suppress distrust around other issues. By examining the relative size of communities over time, we uncover occasional breakthroughs where pro-groups temporarily gain control of specific topic interactions, despite their smaller overall numbers at the 2-topic level (SI Sect. 9).

Figure 4 uses an agent-based simulation to compare the effectiveness of mitigation schemes that target a specific topic or geographic scale versus schemes that blend topics and scales. The model simulates ‘deactivating’ communities by removing their network links, then allows ‘reactivation’ based on connections. In the geography-focused simulation (Fig. 4A, SI Sect. 10), local nodes are randomly ‘deactivated’ at each time step to represent geographically targeted debunking/fact-checking campaigns focused on that locality. Deactivated nodes can then reactivate based on the proportion of global pages they follow. The topic-focused simulation (Fig. 4B, SI Sect. 11) evaluates the impact of single vs. multi-topic messaging. Topics are chosen at a granular level (e.g., “COVID-19”, “COVID-19 and mpox”), and nodes discussing a targeted topic have their discussion suppressed to model the application of topic-specific debunking.

Nodes can then reactivate and resume targeted discussions based on network connections to other active nodes, representing the limited effectiveness of real-world debunking. Specifically, reactivation likelihood is determined by the proportion of a node's connections still actively discussing the targeted topic or locality. This reactivation component, based on ongoing content exposure through network links, captures the stubborn persistence of narratives despite isolated mitigation attempts. Both simulations were performed over 1500 iterations, with results averaged (see SI Sect. 10–11 for details).

In Fig. 4A, a local community's chance of reactivation is determined by the proportion of global communities it follows out of its total connections. This reactivation probability remains static since the geographic global/local status of a community is constant. Conversely, in Fig. 4B, the reactivation likelihood for a community to resume discussing a specific topic is based on the fraction of connections currently posting about that topic. This probability is dynamic, being recalculated at every timestep of the simulation due to the ever-changing nature of topic discussions among communities. Notably, communities that follow only local communities or do not follow any communities discussing the topic have a 0% chance of reactivation. In contrast, those following only global communities or all communities discussing the topic have a 100% chance of reactivation.

We thus find that post-pandemic, the Facebook communities originally focused on vaccines strongly entangle multiple non-vaccine topics and geographic scales both within and across communities. As demonstrated by our simulations, this gives the current distrust ecosystem a unique system-level resistance to mitigations that target a specific topic or geographic scale—which is the case of many current schemes due to their funding focus. The geographic scale simulation (Fig. 4A) shows it is not possible to ‘deactivate’ all local communities with messaging focused solely on the local level, due to reactivation driven by interconnectedness. The topic scale simulation (Fig. 4B) demonstrates superior effectiveness of multi-topic compared to single-topic messaging for reducing discussions, enabled by the entanglement across topics. The curves in Fig. 4B show how the number of communities discussing a particular topic decrease over time due to the mitigation strategy, and the overall trends are represented by averaged reduction curves. Separate curve components that were averaged can be found in SI Sect. 11. The effectiveness of the type of messaging was measured by the rate and magnitude of the decrease in the number of communities over time. Even for two-topic messaging, the proportion of communities discussing a topic decrease to less than 50% in under 600 steps, and further decreases with more topics. Though 3-, 4-, and 5-topic messaging perform similarly, these results show two-topic messaging should be employed by default in future mitigation schemes, as complete knowledge of the distrust web is not required to implement this approach effectively.

## Discussion

Our results reveal new insights into the structure and shifts within the online vaccine distrust ecosystem on Facebook, pointing to potential improvements in mitigation strategies. We analyzed a large Facebook ecosystem of ~ 100 million users focused pre-pandemic on vaccine attitudes. Post-pandemic, their conversations blended multiple topics and geographic scales, conferring system-level resistance to targeted interventions. This highlights gaps in current approaches, which are often constrained by the narrow funding focuses of supporting entities. For example, government-funded efforts typically target misinformation only on certain topics relevant to public health or elections^12,21–38^. Instead, effective mitigation may require “glocal” messaging combining strategic topic and scale mixes. For example, pairing diseases with climate change, or local and national focuses.

Our dataset represents only select languages popular on Facebook^63^, however SI Sect. 2 uses page administrator locations as a proxy to show that our dataset is indeed diverse. Additional sentiment analysis or natural language processing of posts could further enrich insights into the dataset, and of course other social media platforms exist. We note that although our study is technically a large sample of the actual online population, the large number involved (approximately 100 million) suggests it qualifies as a crude population-level map. Indeed, we did not obtain the nodes and links by simple sampling but rather by detection and then following links from node to node. After a while, this tended to return to the same nodes and hence, like circling the globe, hints that we have mapped—albeit crudely—the skeleton of the true online distrust ecosystem. Thus, despite limitations, these initial maps of this ecosystem’s structure and post-pandemic shifts already indicate deficiencies in current mitigation efforts while pointing to alternative strategies worthy of deeper exploration.

### Supplementary Information

Supplementary Information.

## Data Availability

All data needed to evaluate the conclusions in the paper are present in the paper and Supplementary Information (Refs.^40,42,55–59,64–71^). The code used to generate the map in Fig. 1, and from which the results in Figs. 2 and 3 are obtained, is Gephi which is free open-source software. Figure 4 was obtained using Mathematica.
