# Supplementary material for: Rise of post-pandemic resilience across the distrust ecosystem
Source: Sci Rep. 2023 Sep 20;13:15640. doi: 10.1038/s41598-023-42893-6 (PMC10511636; doi:10.1038/s41598-023-42893-6)
Supplement: Supplementary file 1 — Supplementary Information. [file 41598_2023_42893_MOESM1_ESM.pdf]

Supplementary Information (SI)

This SI provides all the information required to understand the main paper, and more—but we feel it is important to include it all since it addresses questions that we have been asked about this social media Facebook analysis/dataset for prior papers upon which this paper also builds. Hence, we are supplying this broader information since any such broader questions left unanswered may leave doubts about the work.

CONTENTS:

Section 1: Methodology:
Collecting data, building network, use of term "glocal", and analysis in the paper.

Section 2: Breakdown of page admin country locations. Color scheme for neutral nodes in network plots. The system before COVID-19, one year later, and three years later. Geolocalized network. Preventing node overlap in Fig. 1 networks.

Section 3: Classification of neutral nodes.

Section 4: Example of Facebook banners promoting best-science Covid-19 guidance. Positions in network of the nodes that receive Facebook banners promoting best-science Covid-19 guidance.

Section 5: ForceAtlas2 layout and analysis showing dependence of layout on strength of bonding.

Section 6: Topic filter. System filtered by topic. System in October 2022 without node labels, and only with those labels appearing in Fig. 1. Fig. 1C–G in the main paper, with node labels.

Section 7: Chi-square test for topic-glocality.

Section 8: Use of nVenn diagrams. General system dynamics, glocal breakdown, and comparison to simulation.

Section 9: Relationship between the number of topics and glocality. Topic heatmaps.

Section 10: Details of the geographic scale simulation.

Section 11: Details of the topic simulation.

Section 1: Methodology:
Collecting data, building network, use of term "glocal", and analysis in the paper.

A list of communities (nodes) and links will be provided online with the Supplementary Information (SI) on publication.

Our **methodology for data collection and classification** follows the 2020 paper referenced in the main text (40). We go further with the classification than in that paper, by sub-categorizing the neutral communities (the "greens", all non-red and non-blue nodes in Fig. 1B) according to their page's declared interest, e.g., parenting community. This is relatively easy since community pages are organized around a particular stated interest. As for the original classifications in (40), this entire process is carried out with the help of three subject matter experts working independently to classify the neutrals ('greens') by interest, and then cross-checking between them for any differences, as discussed below. There were no remaining contentious cases following this process. Here we refer to our research team, including the subject matter experts, simply as "we".

The process is as follows for **building the network of communities** (N.B. each community is a Facebook page). We start with a seed of manually identified Facebook pages discussing either vaccines, public policies about vaccination, or the pro-vs-anti vaccination debate. These were obtained by searching on Facebook's search engine in 2018 and 2019 for key words and phrases involving vaccines. Pages' selection was agnostic to the glocality of usernames, which is to say, there was no concerted effort to select pages with names that explicitly mention a geographical location. At that time, such pages were easy to find. Then we captured the list of outbound links from these pages (i.e., which pages did each of the seed pages recommend to its members at the page level, see below) using Facebook's GraphAPI endpoint for so-called 'fanned' pages. Some of these links went back to pages already in the list, while others did not. We repeated this process two times to obtain a final list of candidate communities (nodes). In the summer of 2021, Facebook removed this feature from its web interface, but as of fall 2021 the page-level links remained available in the background as an endpoint through Facebook's GraphAPI. Personal account information was avoided, as this is not allowed by Facebook's public API terms of service.

We then prune this link list manually since we are aiming at extracting meaningful links rather than the default of a nearly fully connected network with potential links on all possible topics. Specifically, we filtered out unrelated links by manually classifying the pages from the first step by their content and description. In the second step, we filtered candidate nodes using Facebook's built-in filter for page type, a variable descriptor selected by the page administrator, and then classified those filtered pages for content. A detailed description of the classification and filtering process is included below. In short, we only included pages that were talking about vaccines, or pages that weren't talking about vaccines but self-identified as a cause, community, or NGO and were connected within one step to pages active in the vaccine debate. A link from *A* to *B* means page *A* explicitly lists page *B* as one of the pages to which it links, not necessarily because it agrees with page *B*'s content (or actually 'likes' it, using Facebook terminology) but because page *B*'s content is of interest to page *A*. Such a link could have appeared because page *A*'s users noticed it and then recommended it to page *A*'s managers who then established the link. Such a link creates an information conduit feeding content from page *B* to page *A*, likely exposing *A*'s users to *B*'s content -- and it serves as a flag to *A*'s users for them to look at page *B* and perhaps get involved in *B*'s ongoing debates. At each step, we vetted new findings through a combination of human coding and computer assisted filters.

We then take this list of nodes and classify them—based on their content—as being pro-vaccine (blue), neutral (green), or anti-vaccine (red). To do this, we reviewed the page's posts, about, and self-described category. Pro and anti-vaccine classifications required that either (a) at least 2 of the most recent 25 posts dealt with the pro or anti vaccine debate, or (b) the page's title or about section described it as a pro or anti vaccine page. The neutral (green) classification required that (c) either 1 of the most recent 25 posts referred to the vaccine debate, but that they did not explicitly took a side pro or against, or (d) the about section clearly classified the page as a neutral in the pro-vs-anti vaccine debate, or (e) none of the most recent 25 posts dealt with vaccines but the page self-classified as either an NGO, a cause, a community, or a grass roots organization. Most of the neutral nodes (green) appeared in the second iteration of fanned pages as many nodes in the first iteration were more explicitly focused on the vaccine debate. This makes sense since they are more removed from the debate, i.e., they do not actively engage in it as a focus but rather are pages that have become interconnected with the pros and the antis who do. The subject matter experts in our team classified each node independently, and when two disagreed on their suggested classification (which happened approximately 15% of the time, largely if the material was ambiguous) all three reviewers discussed these cases in more depth. Agreement was reached in every case. Our team collecting and classifying the communities (Facebook pages) consisted of analysts who have several years of experience in analyzing and classifying online community content on Facebook and other platforms, being trained through prior work on the content of their online communities in association with establishment health guidance as well as various types of extremism and hate (63, 64). We only included pages in languages understood by the researchers, such as English, French, Spanish, Italian, Dutch, and Russian. Identifying sarcastic or ironic posts, fake news, or troll behavior (55) is a challenging task, even for subject matter experts, and machine learning models can generate realistic vaccine misinformation (65). However, social media communities are quite vigilant in self-policing against bot or troll-like behavior. Thus, the difficulty in measuring the realism and intent of these posts is best left as the topic of another study.

We now comment on Facebook's nomenclature of 'likes', follows and feed, since terms like 'like' can be confusing: 'like' does not literally guarantee liking in the sense of agreeing with, but rather having interest in. The information here is taken directly from online material, which is so common, with similar descriptions in many places, that we do not cite any particular source. The terms "you" and "your" in this paragraph can refer to the entire page. Facebook's website itself also has essentially the same following text, which we borrow from. A (Facebook) like is a person or page who has chosen to attach their name to your page as a 'fan' though this does not necessarily mean they agree with what is being said, rather it is that they have a keen interest in it and want to see content from it. The page will show up in the about section of your account under likes. A (Facebook) follow is a person or page who has chosen to see updates posted by a page on its news feed. If someone or another page follows a Facebook page, it means the content from the Facebook page will show up on their news feed. By default, when you like a page, you will automatically follow it, and this means the content from the page will show up on your newsfeed. The numbers of likes and follows are very close to each but not exactly the same because at some stage, some people who have liked your page may have manually unfollowed you. Your content won't show up on their timeline. We have never seen a significant difference between likes and followers, and the difference is typically a constant factor of about 5%. Thus, this technicality does not affect our conclusions in any way since we have tested adding up to 15% noise and our findings are robust. Finally, we stress again that the Facebook term 'like' should be taken to mean 'have interest in' rather than actually 'like' the content and hence agree with it -- for this reason, communities with very different opinions can end up 'liking' each other since they seek out each other's opposing content to disagree with it.

We now explain our usage of the term **“glocal”** in this paper. In terms of a page's geography, we use “global” to refer to a page that is not tied to a specific location or that specifically has a broad, worldwide focus, whereas “local” refers to a page that is location-centered or focused on a specific geographic area, such as a neighborhood, city, county, state, or country. For example, the page “Vaccine information for Los Angeles County parents” is considered local since an explicit location (“Los Angeles County”) is mentioned, whereas “Vaccine information for parents” or “Global Trends” is considered global since the name implies a worldwide focus. In terms of a page's topic, we use “global” to refer to a page that features a broad-ranging discussion, whereas “local” refers to a page that features narrow and focused topic(s), such as pages that discuss specifically and only “COVID-19”, “COVID-19 and mpox”, etc. In the case of either geography or topic, we use **“glocal”** to refer to a situation where both global and local characteristics occur together.

In this paper, the study period was chosen to span from May 1, 2022, to October 17, 2022, because this included significant events such as the first confirmed case of the 2022 mpox outbreak in the U.S.A. (56), the U.S. Supreme Court's reversal of Roe v. Wade (57), U.S. President Joe Biden signing into law the Inflation Reduction Act (58), and numerous primary and run-off elections in anticipation of the November midterm elections (59). Facebook was chosen as it has 3.0 billion active users worldwide and is the top social network in 156 countries (67). Recent studies have shown that Facebook's in-built community structure is a valuable tool for addressing information needs and supporting decision making (42, 68, 69). Our main unit of analysis is Facebook pages, which aggregate people around a common interest and are publicly visible. While throughout the paper we refer to each Facebook page as a community, we stress that we do not use any ad-hoc community structure inferred from network algorithms. As explained above, our starting point is the ecosystem of interlinked communities around the vaccine health debate just prior to COVID-19 in November 2019. We recall that a link from community (page) i to community (page) j exists when i "likes" or "fans" j (which strictly speaking means that i has an interest in j's content, not that i actually agrees with j), hence indicating a recommendation to all i's members at the page level. A mere mention by a page member of another page is not enough to create a link. These communities can engage in cross-community chatter across different topics, analyze news articles, etc. As previously mentioned, these communities have been classified as pro, neutral, or anti-vaccination, and are part of a larger dataset of communities engaged in vaccination dialogues. Of the full dataset, 83.1% are still active, and 43% are producing topic-related posts. These are the communities that we see in Fig. 1. If we had included all communities that follow those nodes actively generating topic-related content but were not producing similar types of posts, the system would comprise 70.6% of all communities in the full dataset. Nonetheless, we chose to focus on the characteristics of actively involved communities.

In the rest of this section, we provide answers to potential questions and critiques that may be raised regarding our methodology and research in the paper. Though it repeats some of the material above, we feel this is necessary in the context of the potential question being addressed:

We could define nodes and links in another way, and we recognize that our dataset is ultimately an imperfect sample of some larger “correct” network. Any definition of nodes and links—including ours in the present work—can of course be criticized since we are reducing the many attributes of a real system down to the few necessary to build a network. We could instead for example analyze the content of the posts on the pages, identifying shares and URL links of posts from other pages to build a weighted, directed network that captures how users of one page are actually exposed to the content of another page and driven to it. However, this comes with its own downsides: the content may be in different languages and hence not so easy to identify as being shared, and the terminology can evolve quickly (e.g., the use of

slang to avoid attracting the attention of Facebook moderators). This issue is worthy of a study to establish comparative advantages of the two approaches, but such a study is beyond the scope of the present paper. Ultimately, the “best” choice of links and nodes will depend on the questions being asked about the system, since the network is less useful if it has too few nodes and if it is either very sparse in terms of links or too dense with every node essentially connected to most others. The best choice will also depend crucially on the available data and the level of granularity at which this data is reliable. Even with this, the complexity of the data will require some form of simplification in order to make the analysis tractable and understandable. Fortunately, the simplification that we make of each node being a community (page) does bring some advantages: for example, it avoids the need for accessing individual-level information and makes the definition of each node unique since each page has its own unique identification number. Further, as the process we follow yields on the order 1000 nodes (communities), each containing on the order of 100,000 users, the network produced by our definition is visually manageable and yet interpretable at scale, as opposed to being overwhelmed with links or being too sparse. This means that the open-source software Gephi and its ForceAtlas2 algorithm that we use, which follows the principle of energy minimization, provides an uncluttered spatial representation. It further means the network is scalable to the population level, since 1000 nodes with 100,000 users each means we are potentially tapping into the behavior of 100 million users.

We also note that the set of nodes and links is obtained without regard for the specific classifications of the nodes (i.e., pro vs anti vs neutral parenting, etc.). Furthermore, they were obtained without regard for whether or not the node label (page username) explicitly mentioned a geographic location. Thus, the fact that the modular structures of such subpopulations emerge spontaneously in Fig. 1B lends support that the links we identify are meaningful. Taking a devil’s advocate position, if the link methodology were so subjective that the links are not meaningful in a scientific sense, one would expect similar outcomes to a null model, which is not the case.

We have investigated that instead of using human experts, we train a supervised language model of the recent posts of each page, running it over newly discovered communities (pages) and including it in the network only if it can be considered relevant by the model. This gives a crudely similar list—however there are some glaring anomalies that are easily caught by a human expert, showing that while such automation improves volume and speed of analysis, it can also introduce glaring anomalies that a human would be very unlikely to let through. Also, it is hard to have a supervised language model treat material in Spanish, French, Russian, etc. on an equal footing to English. So, although such machine automation sounds desirable because it scales and promises to be less subjective perhaps, it is clearly a trade-off. Overall, the fact that we had already published a similar list of nodes and edges in (40) and the present list—obtained from scratch—was similar, combined with the fact that the number of nodes and links are not too large, and combined with the fact that the network gives results which are very different from a null network model, gives us confidence that our study, though imperfect, is capturing significant features of the actual online system.

Another approach would be for us to list a series of terms we consider relevant, and automatically include communities with sufficient posts using these terms. One might think that this could help ensure that the sampling is more systematic, transparent, and reproducible. However, it has a downside, in that we see the terms evolving in time—in part to avoid attracting the attention of Facebook and in part since the topics are evolving (e.g., the use of bleach then turned to other hot topics) and so such a list could also forever be playing catch-up. Further, there is the prior question of terms translating between different languages. Ultimately, whether this approach would perform well would need to be carefully studied and would represent a research project and paper in its own right.

We stress that we do not assume in any way that linked pages from a given page immediately influence the browsing and information sharing of the users subscribed to the linked pages, and hence that strong correlations in activity levels should accompany links between pages. The neutrals hardly ever discuss vaccines—but the fact that they have material from anti-vax pages appearing in their feed, will be noticed by many of them, and so when some later decision needs to be made such as taking their children to be vaccinated, they may think twice. Thus, it is not that the links carrying material automatically give rise to higher activity at that moment, but that they represent an influence. (55) showed experimentally and theoretically that an online community can suddenly tip to an alternate stance in a reproducible way if there is a committed minority of around 25%. No amount of prior analysis of levels of activity in the experiment of (55) would have predicted this. Having said this, one might wonder if there is some evidence of elevated activity levels. Though this is not important in our paper, we note that we do indeed see this, as shown by the following earlier work of Nicholas Gabriel in our group: consider two Pages i and j, among a collection of N Facebook Pages, and suppose that Page i likes Page j so in our analysis there is a link from Page i to Page j. We calculate a measure of correlation between Pages for unlinked and linked Pages, assigning 1 for a link and 0 for no link. The mean for the unlinked Pages is 0.115 and the mean of the linked Pages in 0.255. Since these samples are relatively large, and their means relatively far apart, this gives an extremely small p-value of p=0.0000037 for the hypothesis that the means are the same. The reason the correlation is not much large is likely that (i) the links are not fully utilized by Facebook's algorithms to "forward" posts to the user feeds, e.g., all posts may not be shown from a linked Page if they are not interesting, or historically they haven't had success sharing along a particular link. In that way the links may be present, and hence the feed read and understood by users of the other community, but not necessarily responded to; (ii) people likely read and digest the content, but do not then go and post new activity in immediate response. In other words, the link has influence on the reader—but this influence likely sits passive until a later time. To extend this analysis, which again goes beyond the requirements and scope of the present paper, one could perform a Granger test of activity time series in pairs of linked and unlinked communities, including the link present as a variable in the times series models and assessing whether it can be considered significantly positive. A second approach would be to retrieve URL links and shares from the posts of all communities and run the test that the frequency of these links from one community to another is higher if they are linked as in the studied network.

One might query analysis and interpretation of the resulting visual shape of the network clusters with the ForceAtlas2 layout in Gephi. But the reason the layout can be interpreted in this way, is that the ForceAtlas2 layout is the result of a many-body physical calculation of energy minimization in which all nodes (regardless of their classification) repel each other with a force that decays with separation, while linked nodes have an additional attractive spring force. Hence sets of nodes that end up closer together do so not because of their hate classification, but because they share more links. It is certainly true that the final layout may be one of many many-body equilibrium states with similar overall energy and hence a local but not global minimum. However, as we show in Sec. 4, angles and lengths can still be used as a crude guide for changes in the network given that the system stays in this local minimum — and it is clear even visually from Fig. S2 that there has been a strengthening of bonds (i.e. sets of links) however one chooses to measure this. In the future, again out of the scope of the present paper, we would operationalize this by developing an optimal network science measure of indirect bonding strength between communities, then performing a statistical analysis versus a null model that shows that there was indeed a reliable structural change between years.

__Section 2:__ **Breakdown of page admin country locations. Color scheme for plots. The system before COVID-19, one year later, and 2.5 years later.**

Color scheme for neutral nodes in network plots:

Table S1: Color scheme for neutral node categories in Fig. 1B

Neutral category	Color
AltHealth	
Conspiracy	
GMO	
Health	
Illness	
Movement	
Organic	
Organization	
Other	
Parent	
Pet	
X	

Breakdown of page admin country locations:

Additionally for interest, it is possible to extract a list of admin top countries (i.e., the country in which the largest number of page admins reside) for all the pages collected. This information was retrieved for 834 of the pages in the dataset, or 62.17% of all pages, and the top 10 countries are as follows:

Table S2: Top 10 counties in which the largest number of a Facebook page's admins reside

Country	Number of pages
United States	352
Australia	33
Canada	27
United Kingdom	20
Italy	9
France	8
New Zealand	6
Sweden	4
Germany	4
Belgium	4

Other countries such as: India, Ireland, Norway, Switzerland, Mexico, Uruguay, South Africa, the Netherlands, Slovenia, Thailand, Malaysia, Brazil, the United Arab Emirates, Croatia, Israel, Austria, Slovakia, Bulgaria, the Philippines, Singapore, Romania, Belize, Denmark, Serbia, Czechia, Portugal, Pakistan, Poland, and Costa Rica also appear in these results, though with diminishing quantities.

These results, however, can only give a partial or incomplete understanding of the true breakdown of admin locations, which a page's Transparency section can shed further light on. The Transparency section

is part of Facebook's effort to provide more information regarding the page and the people who manage it as an effort to increase the accountability and transparency of pages and can be read about in further detail on the Facebook website. Importantly, this information includes the primary country locations where the page is managed and provides a more complete breakdown of where page admins are located.

Some examples of what the Transparency section contains are provided in what follows, with identification information blocked out, and admin information boxed in red. The following page in the upper left-hand corner, for example, would be considered exactly the same as the two, smaller pages on the bottom left and right, which only have admins from one country. Thus, the dataset contains pages where the admins might all come from the same country (e.g., the pages on the right-side of the image and the lower left-hand), but it also contains pages where there is a mix of countries, and that includes countries where the dominant or official language is not English, even if the page's contents are primarily or only in English.

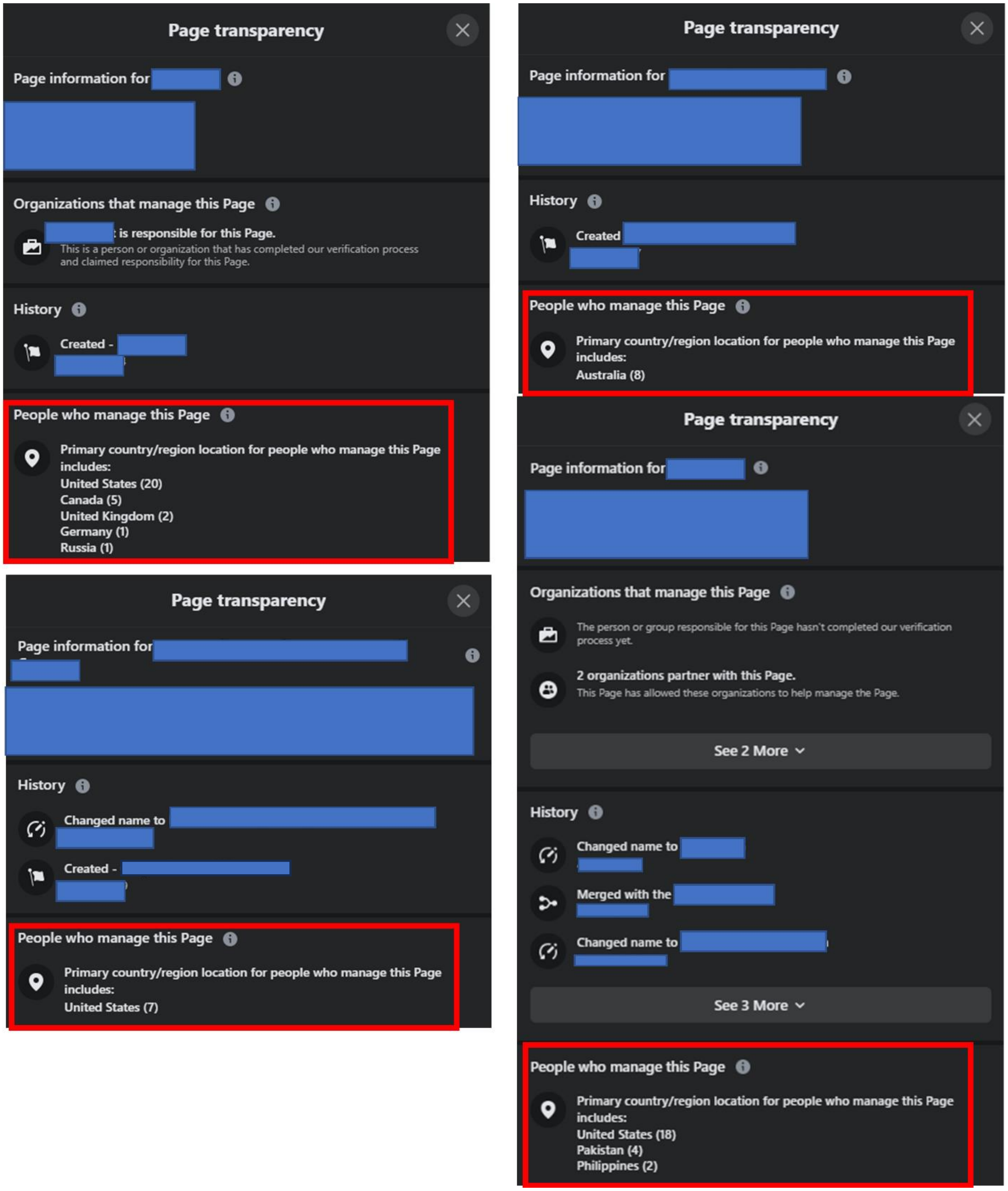

Figure S1: Examples of the Transparency section for several Pages in the dataset. The Pages on the left and bottom right would all be classified as having top number of moderators in the U.S., obscuring in data extraction the true moderation team composition.

The system before COVID-19, one year later, and 2.5 years later.

Similar to Fig. 1 in the paper, this figure below shows what the system looked like at 3 different times: pre-vaccine in Nov. 2019, and post-vaccine in Dec. 2020 and then Oct. 2022. 229 nodes had been removed from this system (deleted) and 35 pages had gone private. The rings provide a visual aid in seeing the increase in node bonding due to the changing node-link structure, and show how similar the system looks post-vaccine as it did pre-vaccine.

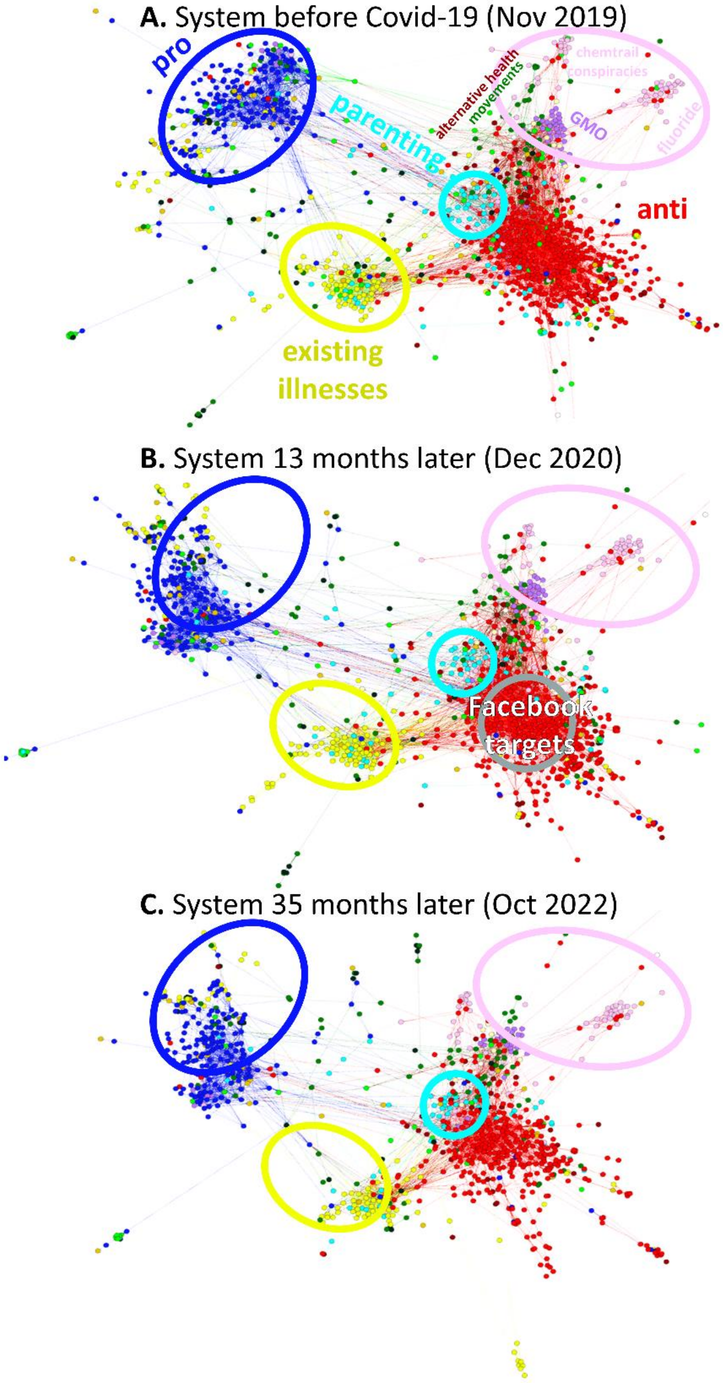

Figure S2: The system before COVID-19, one year later, and three years later

Geolocalized network:

Network of local Facebook communities localized to their real geographic location. Sometimes owners of Facebook pages provide a real address on their page, though in other instances the location has been determined from the Facebook page's username, bio, and other information.

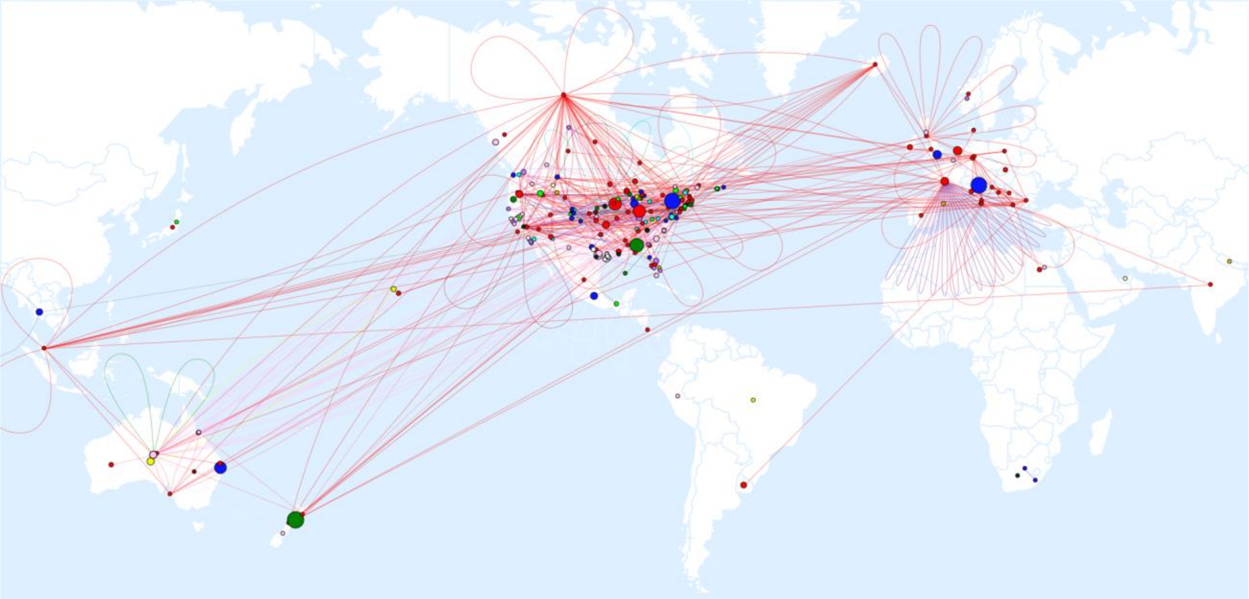

Figure S3: The entire world map with geolocalized Facebook communities; node size is proportional to community betweenness centrality values in full network. Only edges between local Facebook communities are shown.

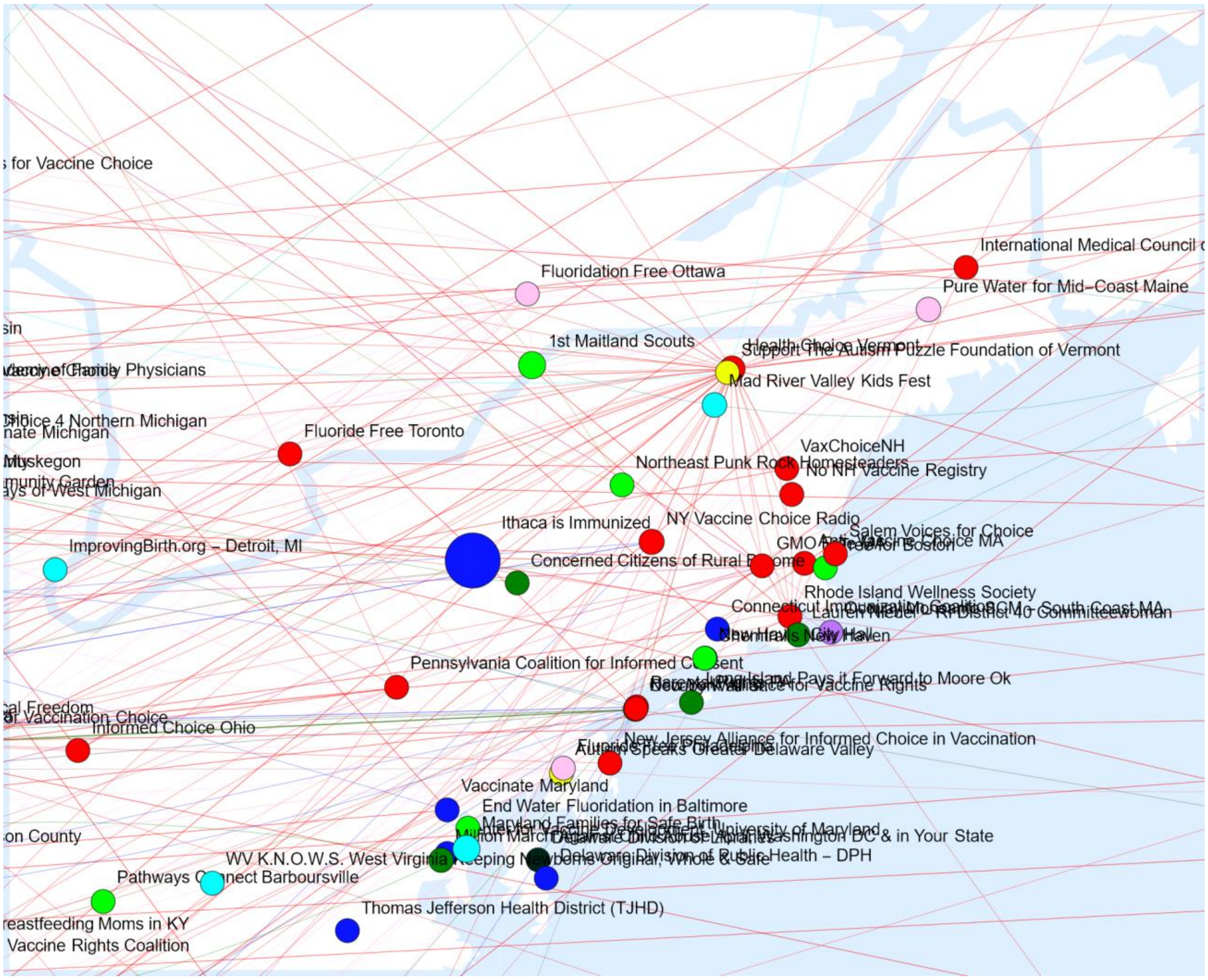

Figure S4: Zoom in on the Northeast and Mid-Atlantic regions of the United States with geolocalized Facebook communities; this is the labelled version of the cut-out in Fig. 1A. Node size is proportional to community betweenness centrality values in full network, and only edges between local Facebook communities are shown.

Preventing node overlap in Fig. 1 networks:

In the main manuscript networks visualized using ForceAtlas2 (Fig. 1), node overlap was allowed in order to preserve the spatial clustering and community structure determined by ForceAtlas2's physics-based force model. However, to address concerns about disambiguating potential node relationships, we generated additional network layouts with the "prevent overlap" feature enabled in ForceAtlas2.

This feature modifies the repulsion force between nodes, so they do not overlap, by taking node size into account when calculating distance. While useful for visual clarity, enabling the "prevent overlap" feature introduces additional space between nodes that can distort the community structures by weakening the spatial grouping of densely connected nodes.

The below figures show the networks from Figures 1A and 1B with the "prevent overlap" feature enabled. Comparing to the main text figures, the general visual clustering patterns are preserved but additional spacing is introduced, particularly in dense sections such as the core anti-vaccine communities. This spacing visually "spreads out" some communities that are more tightly grouped when overlap is allowed. We see then that allowing overlap preserves the spatial clustering that reflects the underlying community structure based on ForceAtlas2's physics-based layout.

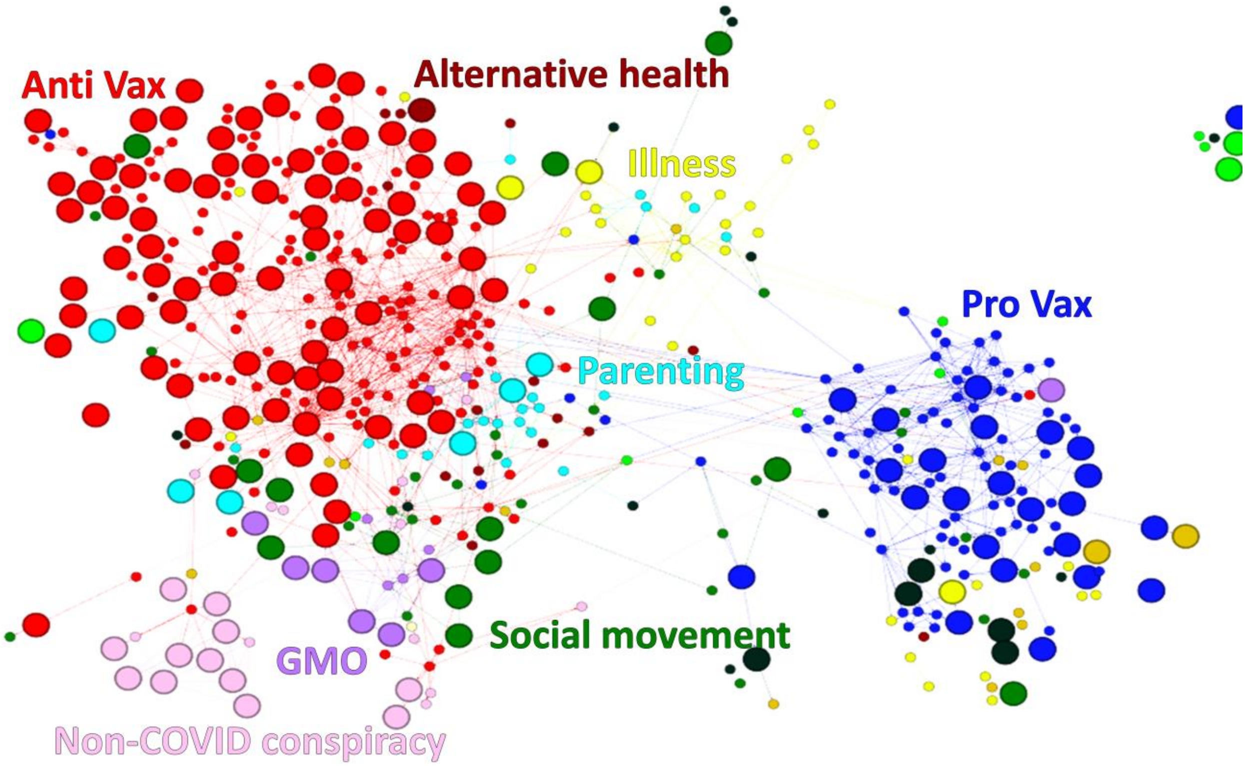

Figure S5: Giant connected component of communities classified according to their stance on vaccines, with ForceAtlas2 layout modified to prevent node overlapping. This is the same as Fig. 1B, but with the "prevent overlap" feature enabled to disambiguate potential node connections. Neutral communities (i.e., non-blue, non-red) are subclassified by their primary interest, e.g., parenting (light blue). Node size indicates geographic scale: large nodes are local communities; small nodes are global ones.

Section 3: **Classification of neutral nodes**

The following pages provide an explanation of the 12 neutral categories of neutral node used in the main paper.

Categories of neutrals

AltHealth

An Alternative Health (AltHealth) community (i.e. node) is a Facebook Page that promotes, discusses, or features content centered around alternative cures and practices, as opposed to traditional medical practical. This includes homeopathy, naturopathy, and spiritual healing. These communities focus on anything from more common conditions such as headaches, indigestion, and general wellness, up to serious illnesses/conditions such as cancer and genetic disorders.

Some of these communities promote and market “remedies” such as essential oils, herbal supplements, or unconventional medicines. They sometimes do this by addressing these products in their posts/pictures and/or sharing links to websites where they can be purchased. Other communities might share anecdotal remedies: this includes recommending alternative diets and practices such as eating “raw”.

While not the largest category in terms of number of communities, this is by far the largest in terms of total user size. This can be attributed to the large size of the top few communities in this category.

For example, the largest green community in the network, “Sun Gazing”, is an AltHealth community.

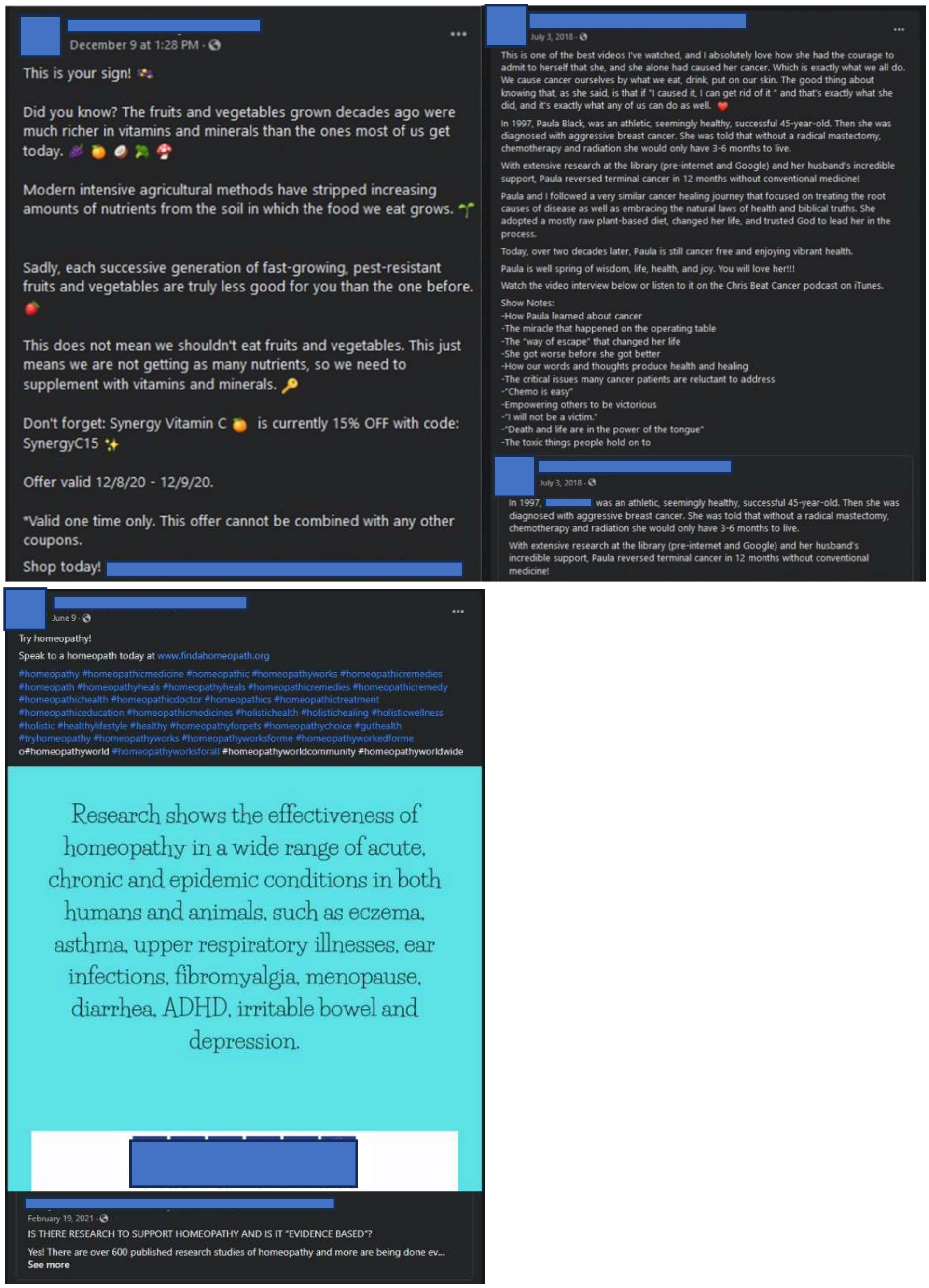

Figure S6: Examples of AltHealth pages

<u>Conspiracy</u>

A Conspiracy community is a Facebook page that promotes or discusses fringe or extreme theories based on unfounded claims that covert actors are responsible for events or circumstances.

This category is dominated by two main conspiracies: fluoride in water and chemtrails. The fluoride conspiracy theory claims governments use it to control the population, lower individuals' intelligence levels, affect fertility levels, and cause health problems. The nomenclature tends to include terms such as

“fluoride free” or “clean water”. Chemtrail conspiracy communities share the idea that planes/aircraft under the direction of the government and shadowy organizations are spraying chemicals in the sky to affect the health and mental capacity of the population below. Although these are two different conspiracies, they share a similar theme of malign actors controlling the population through chemical poisoning.

While there are many Conspiracy communities, they tend to be small in terms of users. The names of these communities tend to include geographical references, such as “Fluoride Free Kansas” or “Chemtrails Global Skywatch of Oklahoma”, which perhaps limit the potential user base.

Nonetheless, these users tend to hold extreme views, which makes them susceptible to other conspiracies. These pages might also serve as a starting point for radicalizing users toward more extreme views.

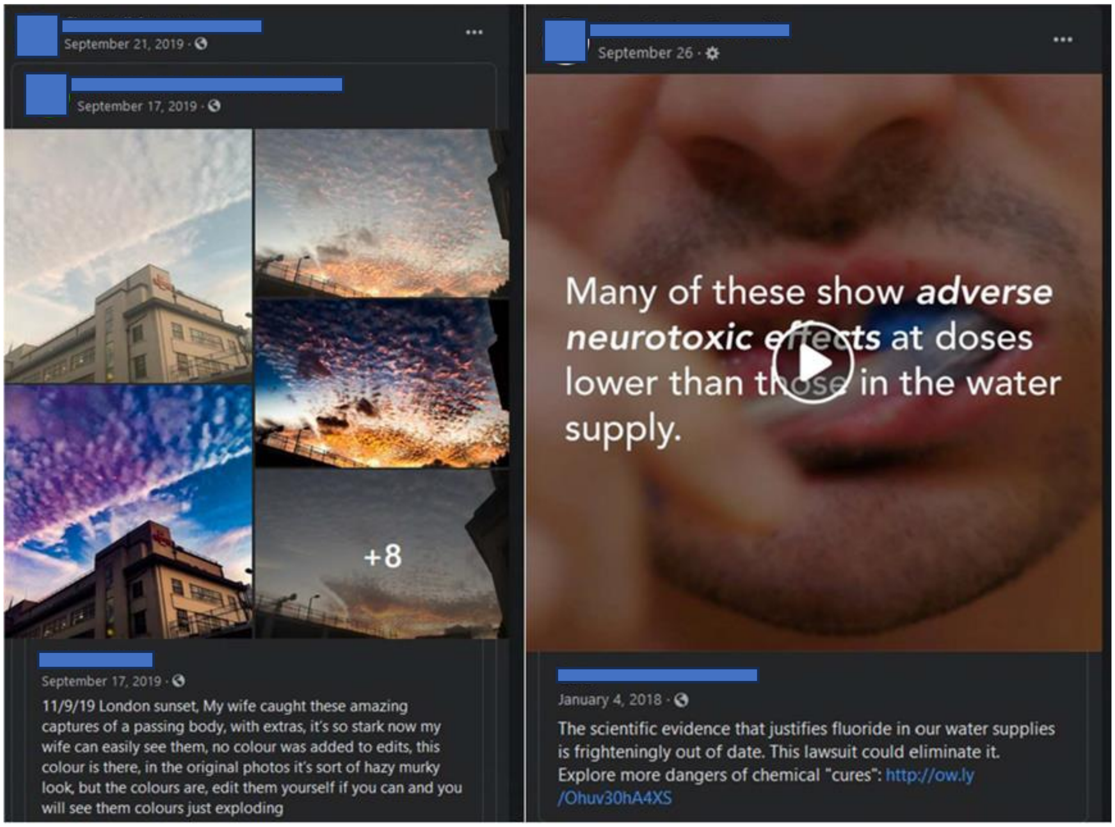

Figure S7: Examples of Conspiracy pages

<u>GMO</u>

A GMO community is a Facebook page that debates or is against the use of genetically modified organisms in food and medicines. Posts generally attempt to raise awareness of products that contain GMOs and argue that these cause harmful effects. These communities often call for a boycott of certain brands or products and/or a requirement to label GMOs. Users often focus on Monsanto as the main antagonist; the company is often named in posts and the names of communities.

There are relatively few GMO communities, and they tend to be small in terms of users. The names of these communities tend to include geographical references, such as "March Against Monsanto Fort Meyers" or "GMO Free Canada", which perhaps limit the potential user base.

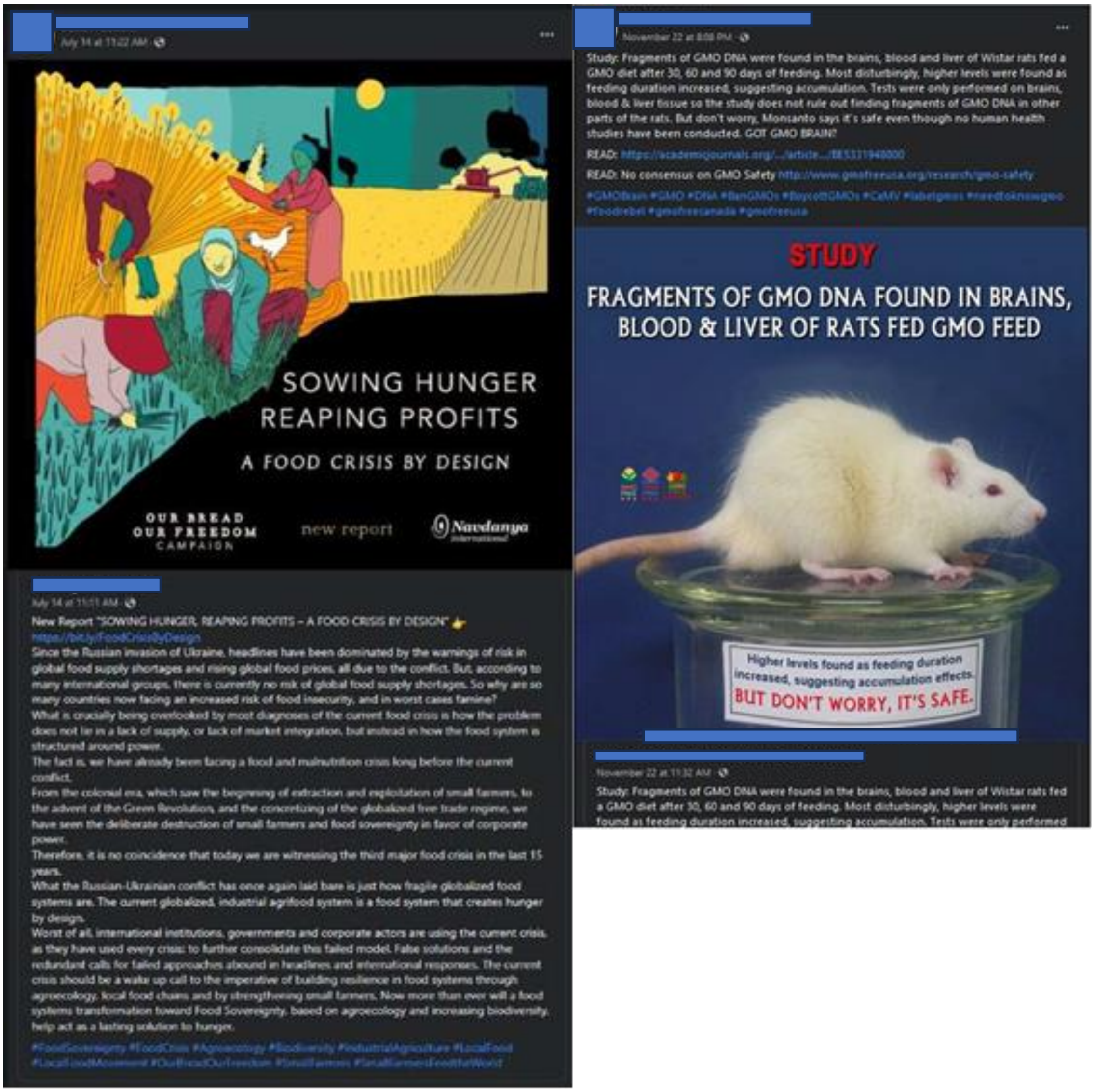

Figure S8: Examples of GMO pages

Health

A Health community is a Facebook page that discusses general health matters and medical institutions. In contrast to the AltHealth communities, the Health communities focus on traditional medicine, practitioners, and institutions. These include clinics, pharmacies, mental health services, medical staff, and health initiatives. The average Health community has relatively few users.

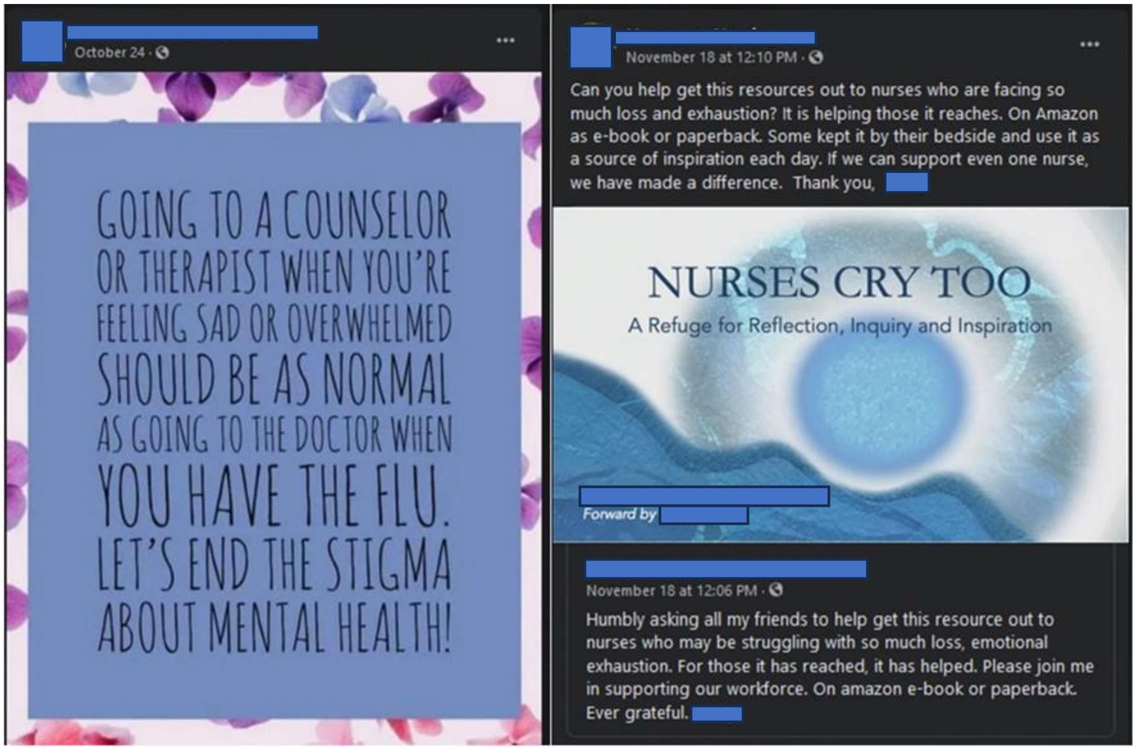

Figure S9: Examples of Health pages

<u>Illness</u>

An Illness community is a Facebook page that aims to raise awareness of, discuss, or serve as a support group for certain medical and mental illnesses/conditions. Fibromyalgia, cancer, and HIV/AIDS play a significant role in this category, but by far the most discussed condition is autism.

Due to the serious nature of these medical conditions, the role of these pages as support groups leads to an interesting dynamic. This is the most common category of green cluster in the network, perhaps because they tend to narrowly focus on a specific illness. Nonetheless, the total number of users in this category is toward the middle of the pack, which means that the average number of users per Illness community is relatively small. These are cohesive communities, featuring discussions that are highly salient to a small number of users. They have relatively active discussions and are made up of users who have the condition or who have family and friends who are affected.

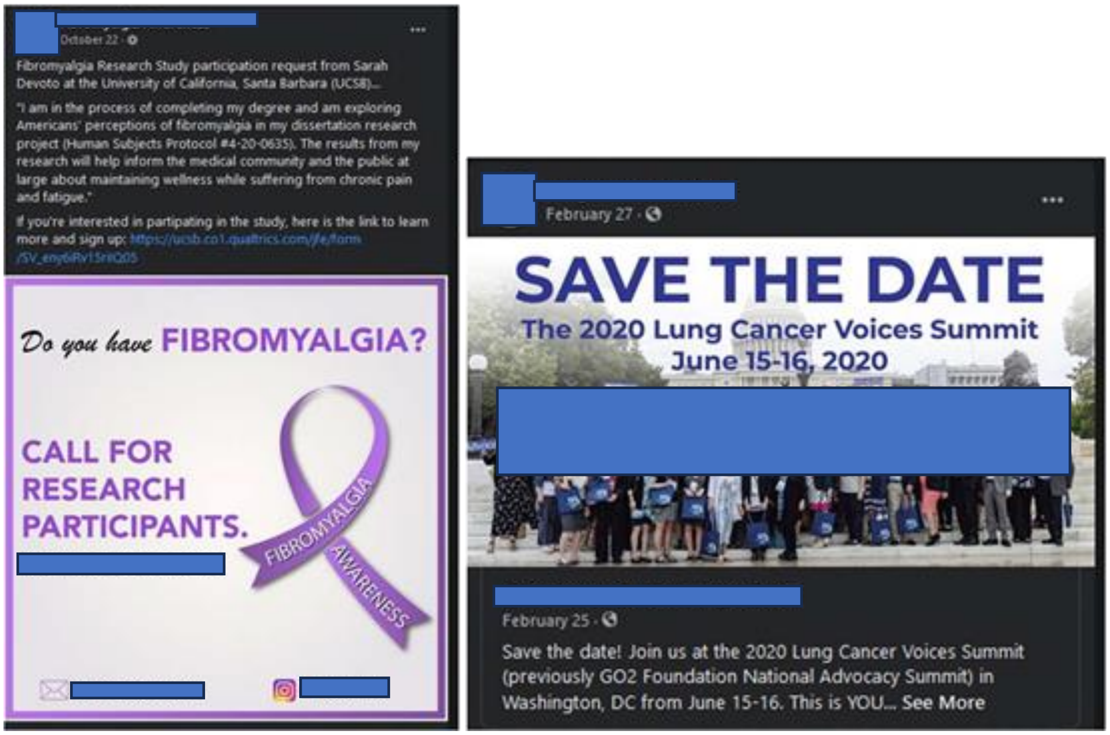

Figure S10: Examples of Illness pages

<u>Movement</u>

A Movement community is a Facebook page that advocates for a specific cause or political objective. The main topics include cannabis legalization, universal equality, domestic violence/human trafficking victim protection, the environment, and mental health awareness.

In terms of both total number of nodes and user size, Movement is the second largest. The wide variety of causes tends to increase the total user size, but not all communities agree with each other. Some even hold opposing views on specific issues. Two of the three largest communities advocate for the legalization of cannabis. Victim protection and awareness is also a common theme among many in this category, including victims of bullying, domestic violence, human trafficking, child abuse, and mental health issues.

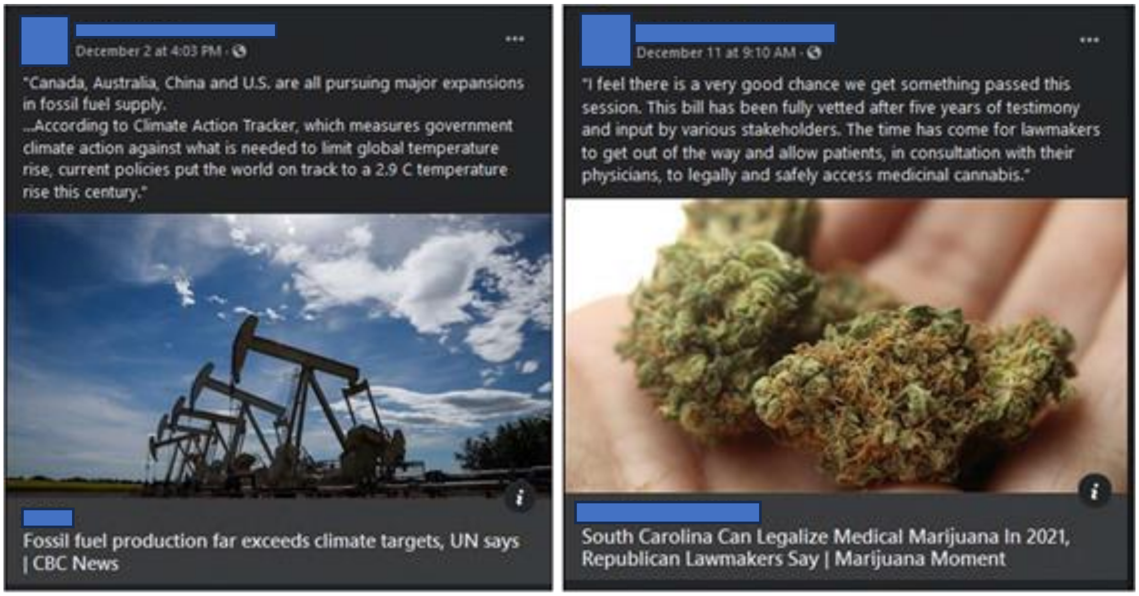

Figure S11: Examples of Movement pages

Organic

An Organic community is a Facebook page that promotes an organic diet and lifestyle. Users tend to share recipes, diet plans, and information about particular organic ingredients. Growing your own food is an important activity that most of these pages advocate.

While this category is the smallest in terms of the number of communities, in terms of total users it is twice as large as the GMO and Health categories, indicating that the Organic communities are fairly popular.

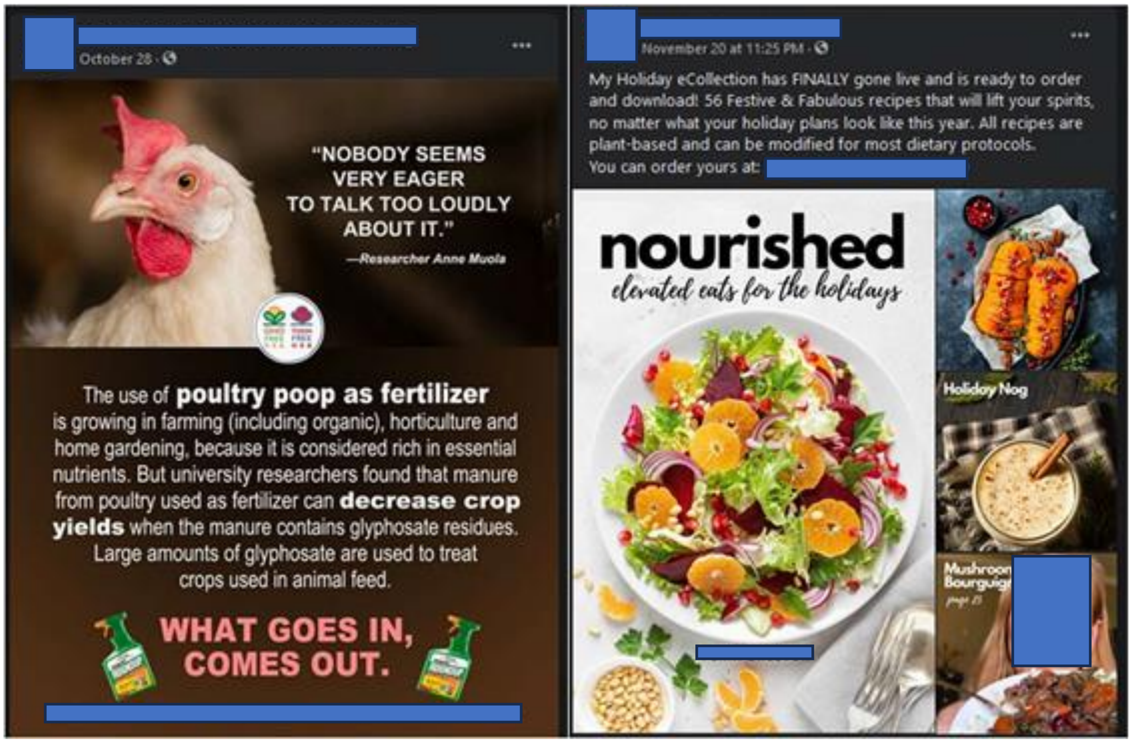

Figure S12: Examples of Organic pages

<u>Organization</u>

An Organization community is a Facebook page focused on a formal institution, including both government and non-governmental institutions. Communities include the U.S. government's Stop Bullying program, foreign government institutions, assistance projects, TEDx Change, and county initiatives such as "Get Healthy Knox County" and "Pathways Connect Central". The largest Organization communities are news organizations, such as the American Independent and LGBT News.

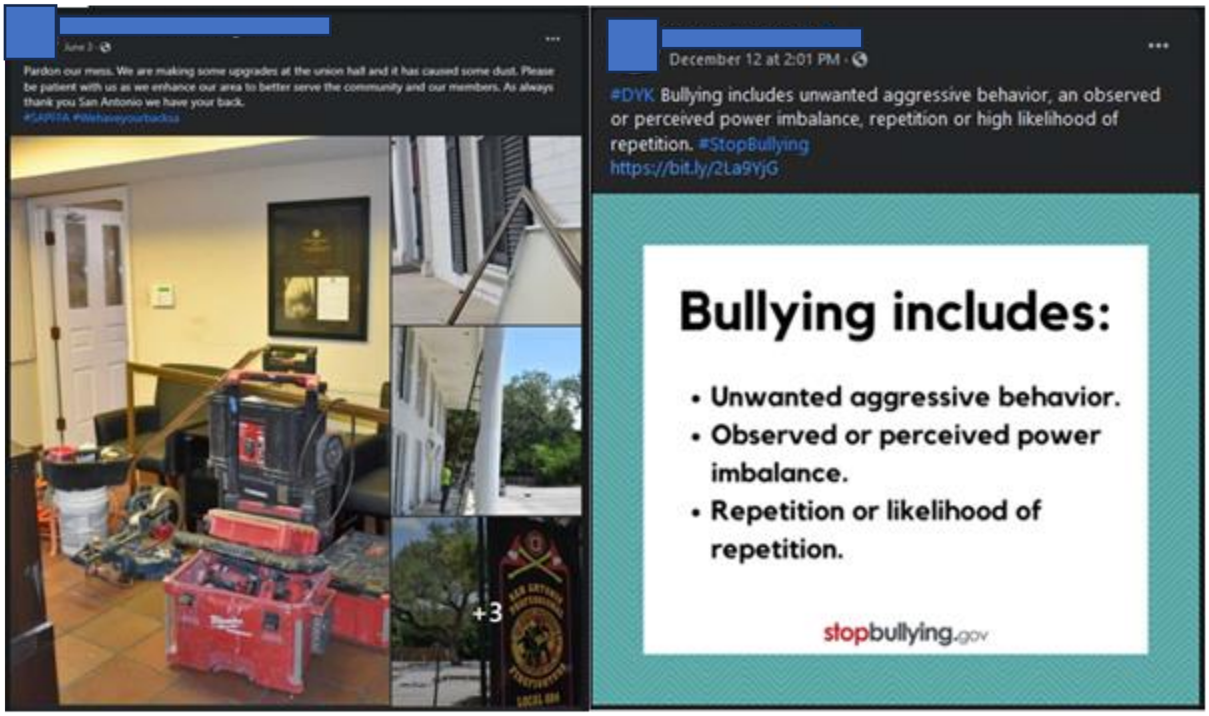

Figure S13: Examples of Organization pages

<u>Parent = “Parenting”</u>

A Parent community is a Facebook page that discusses or offers advice and support for parenthood. The most commonly discussed issues include parental rights, breastfeeding, homeschooling and birthing, and raising children with special needs. Although there are communities focused on issues particular to the role of fatherhood, most of these communities seem to be focused on motherhood and frequented mostly by women.

There are relatively many Parent communities, and they tend to be relatively large, making this one of the most important substantive categories in the network.

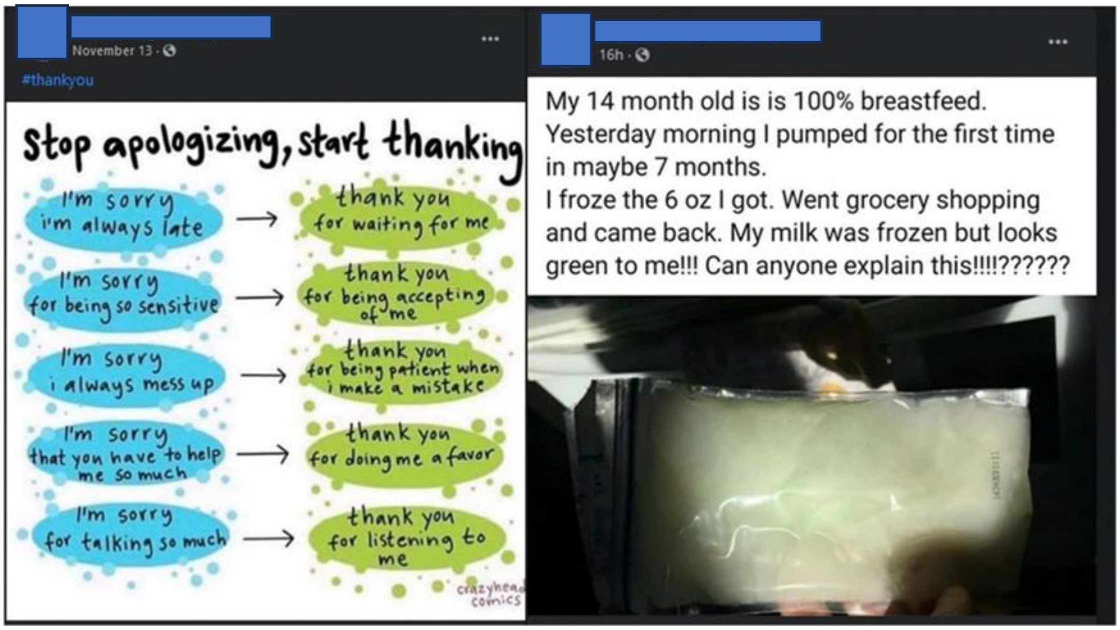

Figure S14: Examples of Parenting pages

<u>Pet</u>

A Pet community is a Facebook page that is centered around pets (typically dogs and/or cats). The category is almost exclusively made up by shelters, pet rescue/adoption, and lost-and-found organizations. A substantial share of these clusters are based in Texas.

There are relatively many Pet communities, and they tend to be relatively large, making this one of the most important substantive categories in the network.

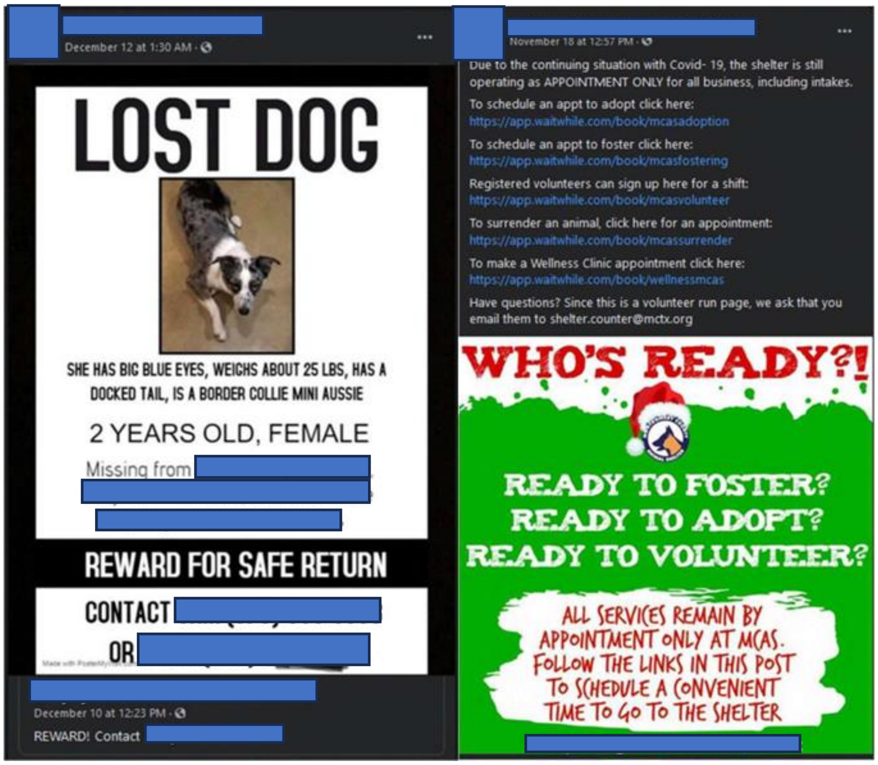

Figure S15: Examples of Pet pages

Other

Some communities did not fit in the categories we created, and thus we classify them as Other. These include communities focused on specific churches, spiritual issues, farming, meme sharing, and community-building. The largest of these is an "earth lover" page, which shares photos of beautiful landscapes and natural features. There are also two large spiritualism communities that tend to share inspirational sayings and general spiritual content.

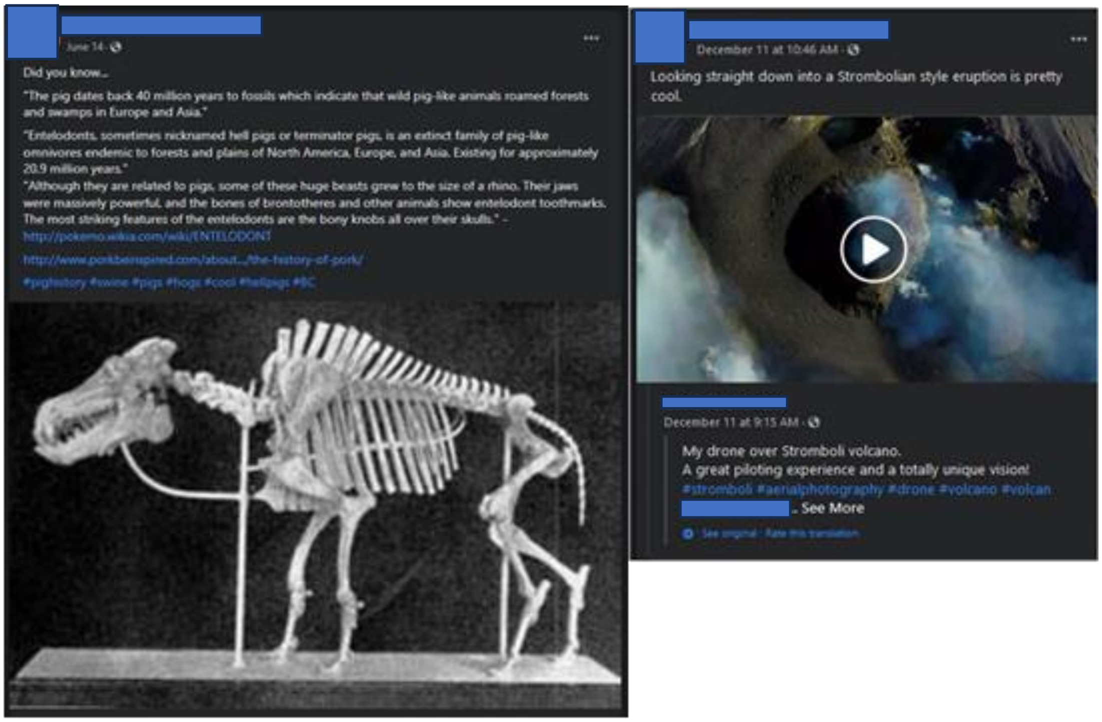

Figure S16: Examples of Other pages

<u>X</u>

X are communities that did not have an interest focus, or it was too ambiguous. They are a minor, unimportant category in our study.

Section 4: **Example of Facebook banners promoting best-science Covid-19 guidance. Positions in network of the nodes that receive Facebook banners promoting best-science Covid-19 guidance.**

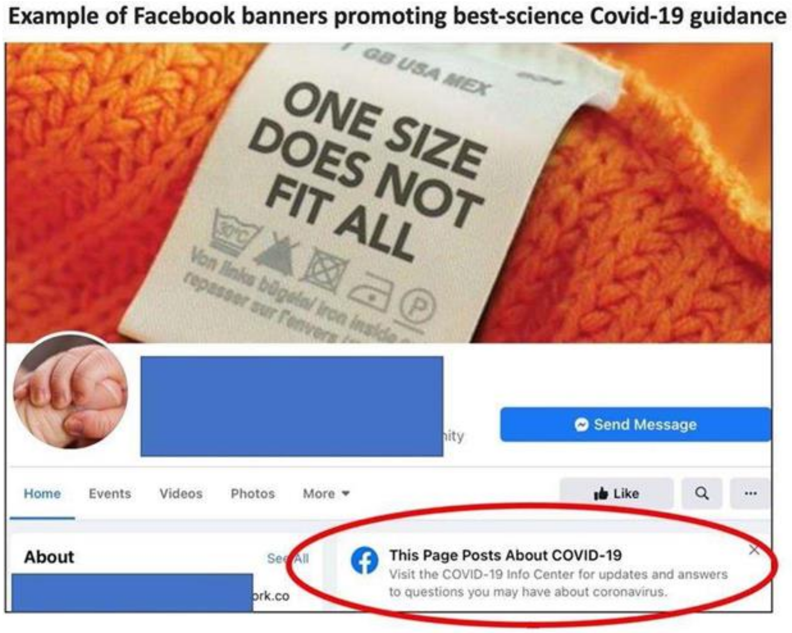

Figure S17: Example of Facebook banner promoting best-science COVID-19 guidance

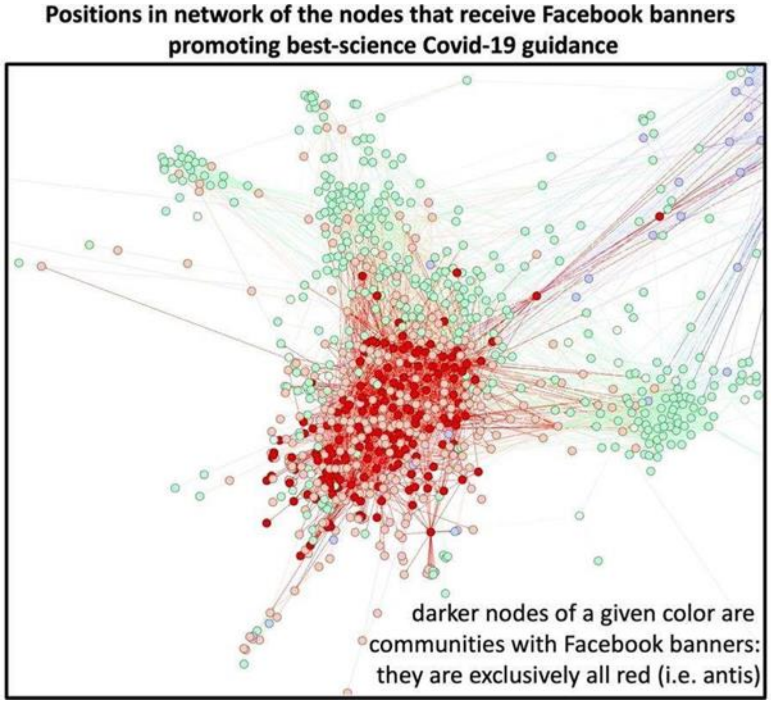

Figure S18: Category and location of nodes (i.e., Facebook pages) that received a banner promoting best-science COVID-19 guidance. Also circled in grey in Fig. S2B

<u>Section 5:</u> ForceAtlas2 layout and analysis showing dependence of layout on strength of bonding.

The ForceAtlas2 layout of Gephi simulates a physical system: it runs a large-scale energy minimization computation in which nodes repel each other while links act as springs. It is color-agnostic, that is, the color segregation and hence segregation of community types in Fig. 1B–G emerges spontaneously and is not in-built. The network nodes and links were built without regard for this color segregation. Nodes that appear closer to each other have local environments that are more highly interconnected, whereas nodes that are far apart do not. Each node (Facebook page) directly receives the feed of narratives and other material from that page and all members (fans) can engage in the discussions and posting activity. One could go ahead and measure bond lengths as the distance between the centers of the respective community types and the bond angle as the angle in between—which can be done in practice by arranging the networks, as we do in Fig. S2, for the two times on the same scale, so that the distance between the centers of the red and blue communities remained the same. Changes can then be measured relative to this, e.g., manually or using the freely available software Aequo.

We now show that changes in the positions in the ForceAtlas2 layout, and in particular visual strengthening of bonding, is associated with changes in the effective link strengths—and hence that sets of communities (nodes) appearing closer together are more interconnected and hence likely have more shared content and users. We use the simple 3-body setup and notation shown in the diagram below:

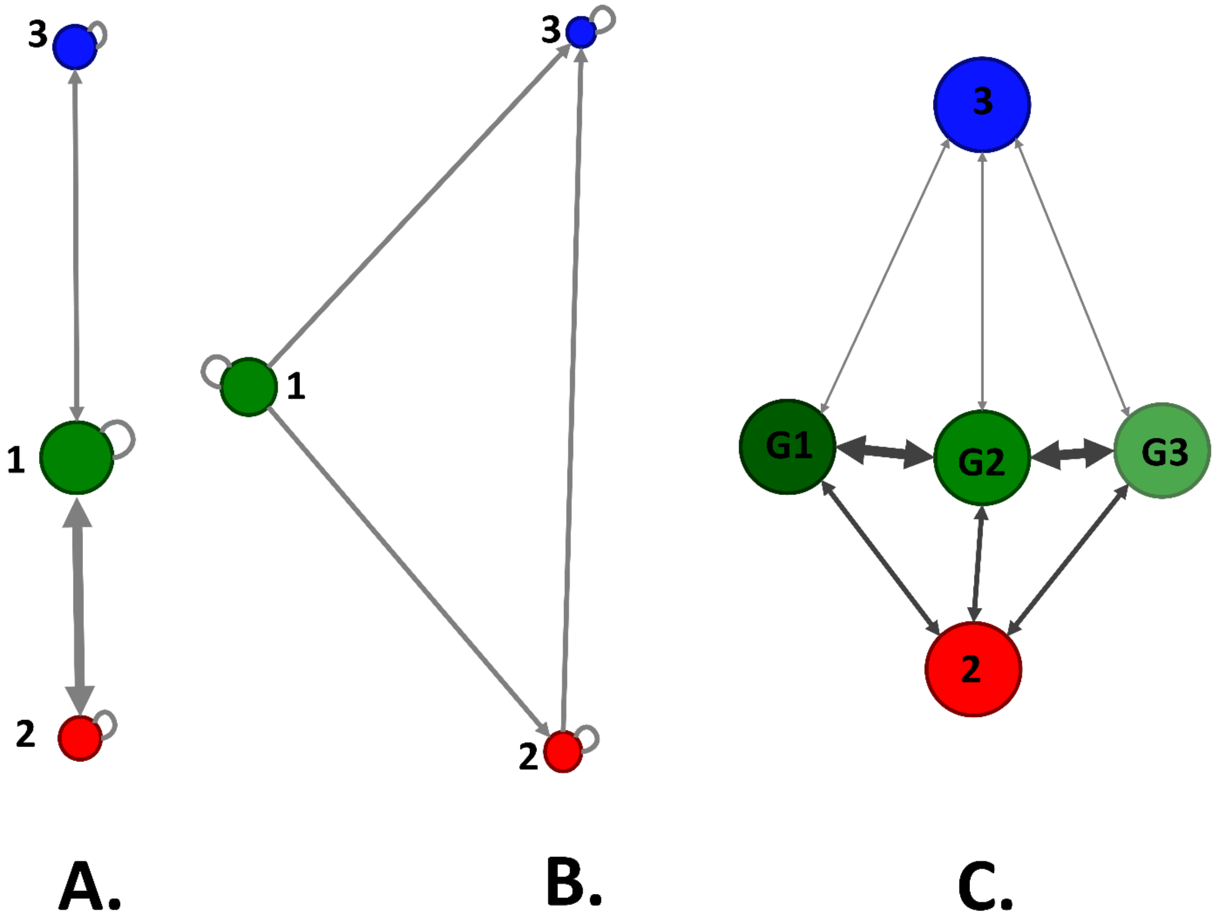

Figure S19: 3-body diagram of systems where nodes represent clusters of communities

Each ball shape in this figure (which can be thought of as a super-node) can represent a coarse-grained version of a collection of reasonably tightly bound balls (i.e., nodes as in the main paper) of the same color, and hence represents clusters of communities of the same type as observed in Fig. 1B in the main paper. In this way, the 3-body analysis that we give below, can represent the much more complex multi-node system in Fig. 1B, simply by renormalizing what a ball is and hence means. For example, the parent communities roughly correspond to position 1, as does the center of mass of the green communities from our 2020 vaccine paper, while 2 represents the anti (i.e., red) communities and 3 represents the pro (i.e., blue) communities. By inputting relative values for the links (equivalently for ForceAtlas2 algorithm, this can be the number of links) between 1, 2 and 3, and letting the ForceAtlas2 algorithm relax the network through energy minimization as in Fig. 1, we explore below what relative weights are needed in the links in order to obtain different arrangements. Our findings are as follows. We consider for simplicity unidirectional links, i.e., 1-2 is a link from 1 to 2, but the results are the same if we use bidirectional links:

- with link weight (or equivalently, the relative number of links) from 1-2 taken as being similar to 1-3, and both much larger than 2-3 (e.g., 10,10,1) then the layout is similar to case a above with bond length 1-3 similar to 1-2;
- with link weight (or equivalently, the relative number of links) from 1-2 taken as being similar to 1-3, and both somewhat larger than 2-3 (e.g., 3,3,1) then the layout is an isosceles triangle as in case b with bond length 1-3 similar to 1-2 and bond length 2-3 larger;
- with all 3 link weights (or equivalently, the relative number of links) taken as being similar, i.e., 1-2 similar to 1-3 and 2-3, then the layout is an equilateral triangle with all 3 bond lengths similar;
- with link weight (or equivalently, the relative number of links) from 1-2 the largest, and 1-3 similar to 2-3 (e.g., 3,1,1) then the layout is an isosceles triangle but now with bond length 1-3 similar to 2-3 and both larger than bond length 1-2.

This can of course be extended to include different categories of neutrals separately, as shown in case C.

Based on this, we can now understand quantitatively why the networks in Fig. 1B of the main paper have clusters of communities in the particular layout positions that they do. To demonstrate this, we take the total number of links from the data between red communities and blue communities, between green communities and blue communities, and between green communities and red communities, and convert them to a relative weighting which is given by the ratio 1: 6.7 : 14.1. Putting this into the ForceAtlas2 algorithm produces a layout as in case a with bond length 1–2 = 3.8 and bond length 1–3 = 6.0 which compares favorably to the empirical results for the layout and distances between the center of masses of the red communities, the green communities, and the blue communities, which also approximates case a and has bond length 1–2 (i.e., Green–Red) = 3.4 and bond length 1–3 (Green–Blue) = 7.3.

Obviously the many-body nature of the actual network, with many nodes and complex link arrangements, introduces the relatively small differences. This analysis also allows us to predict the tipping point where the entire ecology will flip into an arrangement like an equilateral triangle. Though obviously crude, it predicts that this will occur if the total number of links between green communities and red communities decreases by 53% or the total number of links between green communities and blue communities increases by 112%. It also explains why the conspiracy communities sit to one side, based on the same approach of summing the relevant number of links. We note that in all cases, the number of self-links, i.e., green community to another green community etc., are so large, that green communities act as an approximately single entity with its own center of mass (like molecules in a green ball), and the same for the red communities separately, and for the blue communities separately.

<u>Section 6:</u> Topic filter. System filtered by topic. System in October 2022 without node labels, and only with those labels appearing in Fig. 1.

To identify COVID-19, mpox, abortion, elections, and climate change dialogues, we developed word filters that combined post content, post descriptions, image descriptions, and link text, while ignoring cases. For each topic, we created a list of search terms, including equivalents in other languages, such as “aquecimento global”, “globale erwärmung”, “calentamiento global”, “calentamiento mundial”, “réchauffement climatique”, “riscaldamento globale”, “riscaldamento climatico”, and “surriscaldamento climatico” for "climate change". We used regular expressions to catch misspellings, punctuation, and non-English languages. For example, “corona virus” became “(c|k|[(])+orona(no|[[:punct:]]|\\s){,4}(virus\\>|vírus)”. Additionally, we included emojis, specifically the monkey emoji, as often users would attempt to self-censor or evade detection by employing the usage of such terms as “🐒pox”. Word filters were augmented via searches on various social media websites, and identifying how users may try to self-censor their posts. Our flexible approach thus captured deliberate misspellings and word replacements, ignored punctuation, dealt with added spaces in-between the words to avoid filters, and other languages.

Our word filters have limitations. Although we can include various languages, we are restricted to those our researchers can read, potentially resulting in missed insights from other languages like Bengali, Hindi, Indonesian, Vietnamese, and Arabic. Furthermore, machine word filters have a "Scunthorpe problem" as they struggle to understand words in context, which makes it challenging to create a filter that can determine the intent of the text. For example, early in the coronavirus pandemic, “China flu” was coded language to refer to COVID-19, however a filter for “China flu” will also determine posts discussing “China flu season” or “Chinese flu vaccine” as containing COVID-19-related content. Thus, developing such a filter that can determine the intent of the human behind a social media post is a complex task that requires further research, and best left as the subject of another paper.

Fig. 1A shows a portion of the network, with examples of local and global community names enclosed in boxes. Global or worldwide usernames are boxed in purple, while countries are boxed in green, states in blue, cities in red, and regions like counties or neighborhoods in orange. This represents the large online universe that consists of over 4 billion Facebook users, from which we sampled the communities that actively post about one or more of the topics being studied. Fig. 1B is a magnification of the giant connected component of this system, consisting of 100 million users and only those communities that are actively posting. The communities are shown as nodes colored according to their vaccination debate status, with vaccine categories labeled next to where that category is primarily clustered. The size of the nodes indicates the geographic global/local aspect of the community's username, with larger circles representing local communities and smaller circles representing global communities. We can see that the anti (red) and pro (blue) communities are primarily clustered on different poles in this network, with many neutral communities (non-red and non-blue) clustering near the antis. By examining Fig. 1B, we can quickly see that there are only a couple of local Illness nodes, the Anti core is predominantly comprised of local nodes, local Conspiracy nodes cluster together, and there are fewer local Parenting nodes when compared to global Parenting nodes. A detailed breakdown of the system at three different timepoints can be found in SI Section 2.

Pie charts can be used to illustrate the topics of engagement for each node in the network represented in Fig. 1B, and the complete network is displayed in SI Section 6. Figs. 1C–G provide close-ups of various segments. Fig. 1C zooms in on an outcropping of Anti nodes, where "abortion" is a minor topic compared to other topics. In Fig. 1D, which focuses on the Illness communities, only 3 out of 36 communities in the neutral category are local, while 36.4% of antis are local. Interestingly, climate change is the most popular

topic among the magnified Illness communities (88.9%). Fig. 1E magnifies pro groups and reveals that only 24.1% of communities are local, and there is a good mix of topics. Similarly, Fig. 1F zooms in on parenting communities, where a variety of topics are also mixed. In Fig. 1G, portions of conspiracy groups are magnified, and surprisingly, the topics of abortion and election appear to be very popular in this cluster, of which 50% are local. Nearby GMO groups also have a high percentage of local groups (53.8%). While 25.2% of communities are local in the entire dataset, 31.7% of communities are local in the network of only content producers in Fig. 1B. COVID-19 and climate change are the most discussed topics among pros, at 75.9% and 60.2%, respectively, while mpox, abortions, and elections are discussed by 42.6%, 28.7%, and 25.9%, respectively. Examining the network in this way can provide insight into how the topics of engagement relate to the community itself and its position in the system, and suggest that local communities drive potentially divisive discourse in unexpected ways.

Even though Facebook has removed some large and active extreme communities (nodes), operationally the ecology remains largely unchanged because of a hidden self-repair effect. (70) shows there was negligible impact on the subnetwork of top 20 nodes ranked by highest betweenness centrality, which is a measure of a node's (community's) ability to act as an efficient conduit of content. Red nodes (anti-vaccination communities) are the most prominent and highest ranked within these top 20: while many were removed by Facebook, there was a system rewiring such that other red nodes (anti-vaccination communities) took their place. This means that there is a self-repairing core 'mesh' of extreme anti-vaccination communities within the full ecology that have—and can continue to—share and distribute extreme content not only with each other but also with the other nodes in the full ecology, including the millions of users in mainstream neutral communities with whom they are connected, e.g., parenting communities.

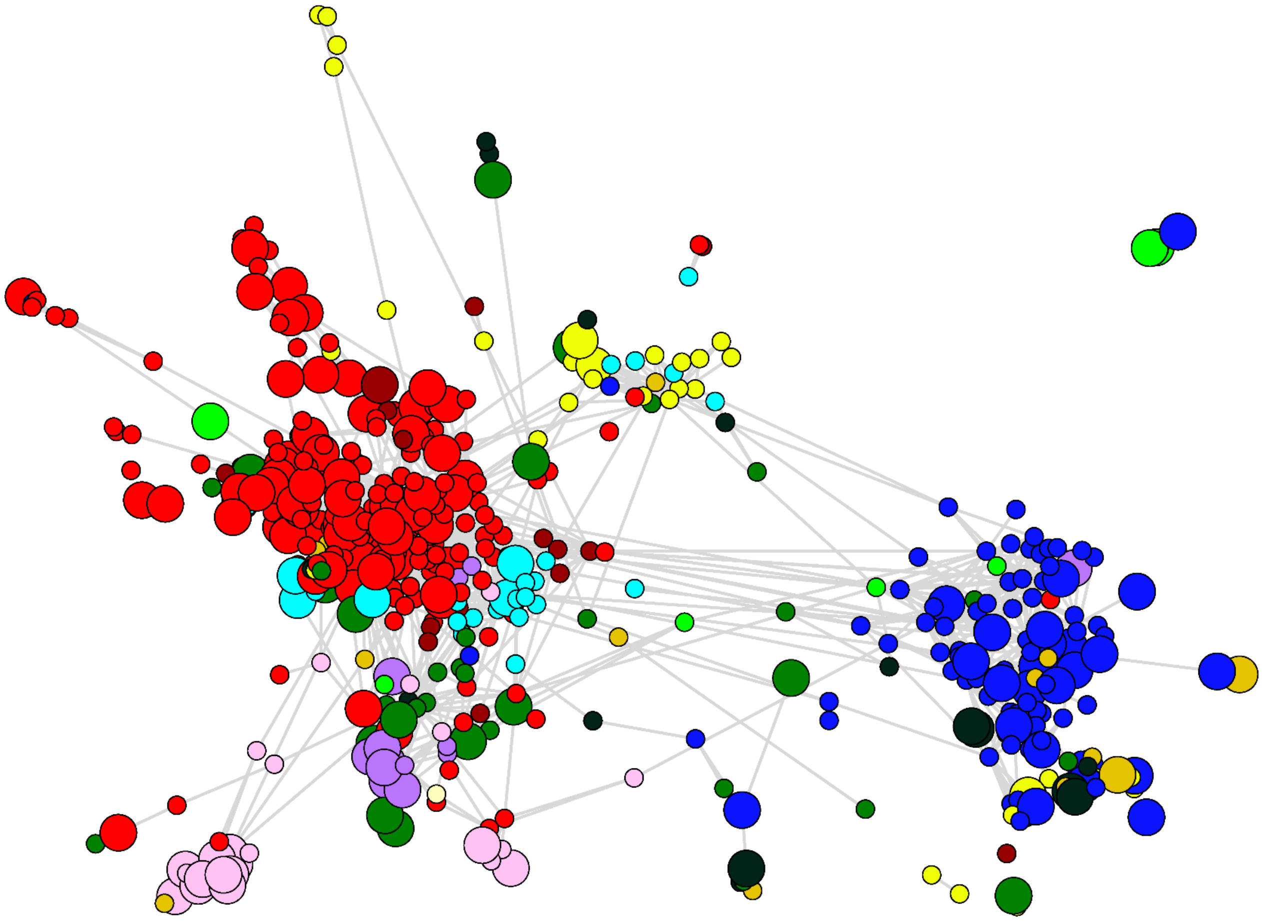

Figure S20: The system in Oct. 2022 is here filtered to only display nodes that discussed at least one of the topics, and the nodes are color-coded according to their vaccine category. This is the network that we use in Fig. 1B.

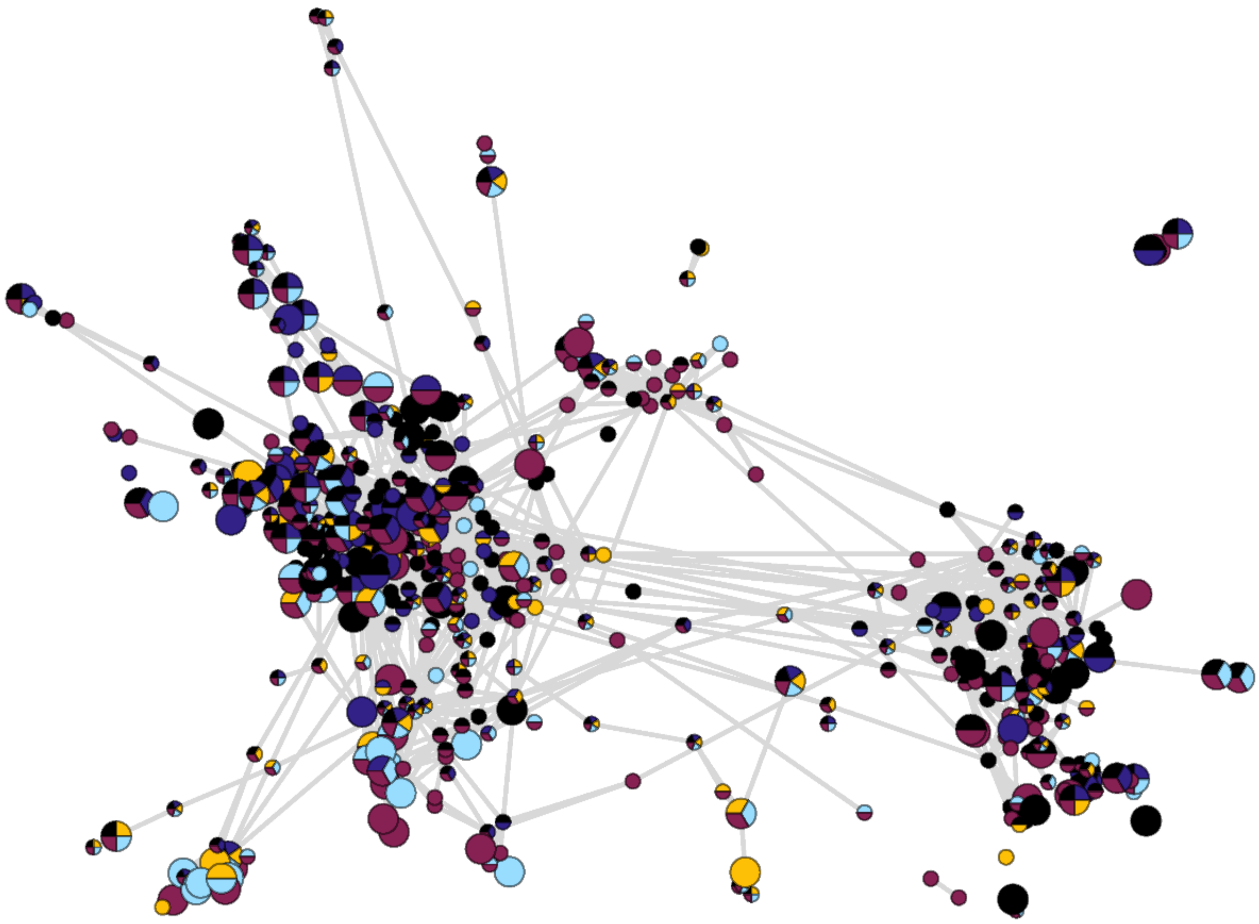

Figure S21: As in Fig. S17, the system in Oct. 2022 is here filtered to only display nodes that discussed at least one of the topics. The nodes are now color-coded according to the topics they cover.

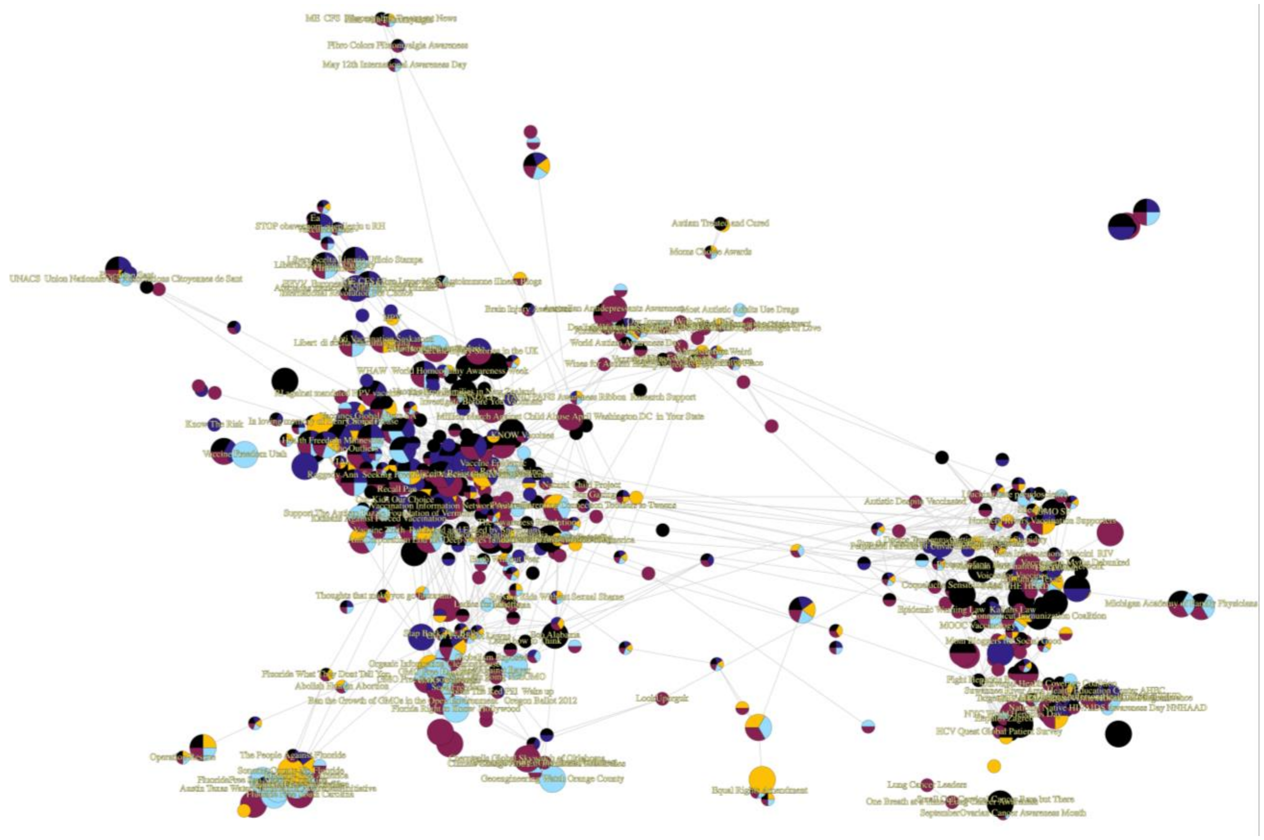

Figure S22: As in Figs. S17 and S18, the system in Oct. 2022 is filtered to only display nodes that discussed at least one of the topics. This network is the same as Fig. S18 but now with node labels added. This is the network used in Figs. 1C-G.

Fig. 1C–G in the main paper, with node labels:

C.

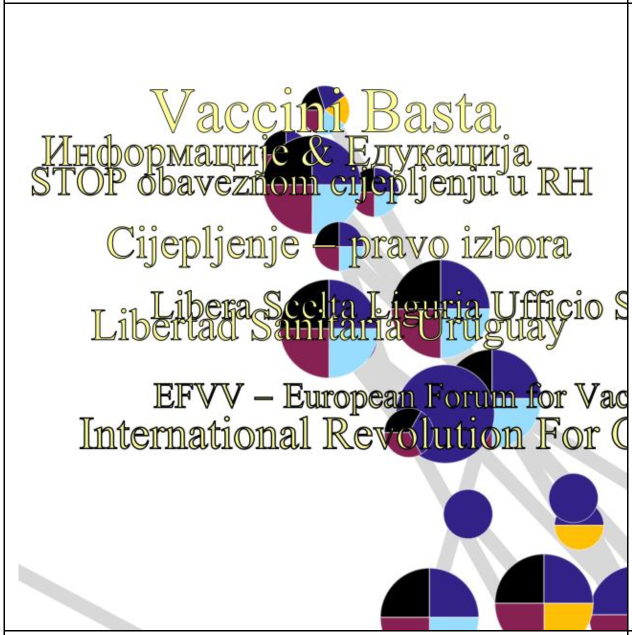

F.

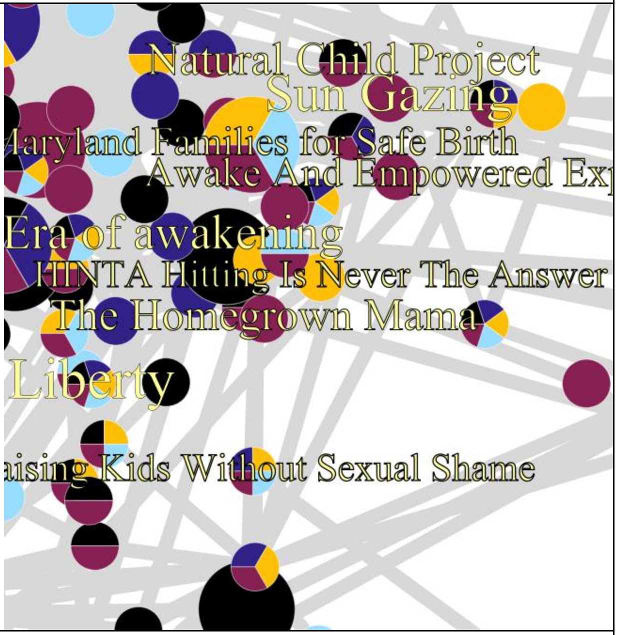

D.

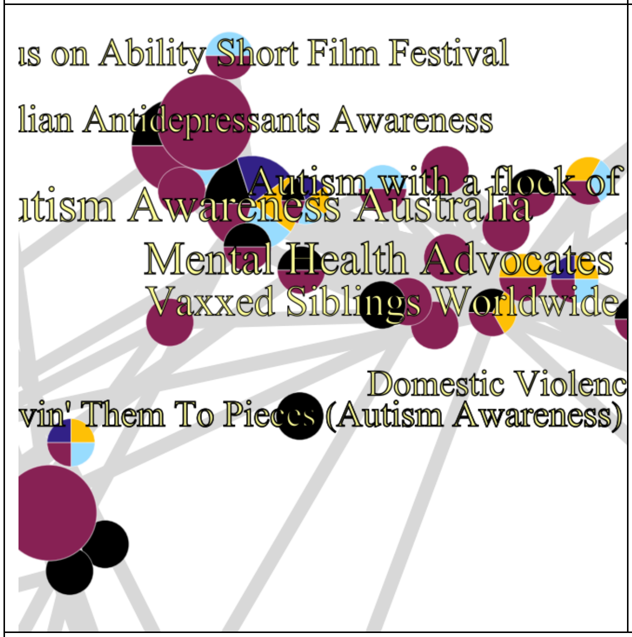

G.

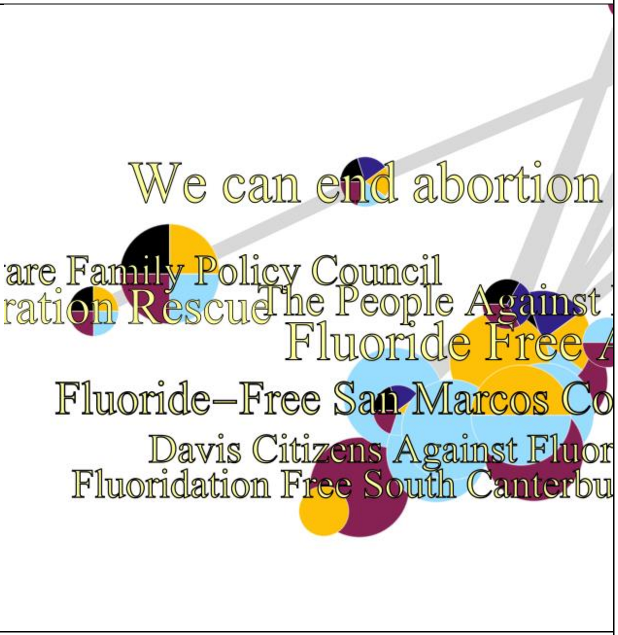

E.

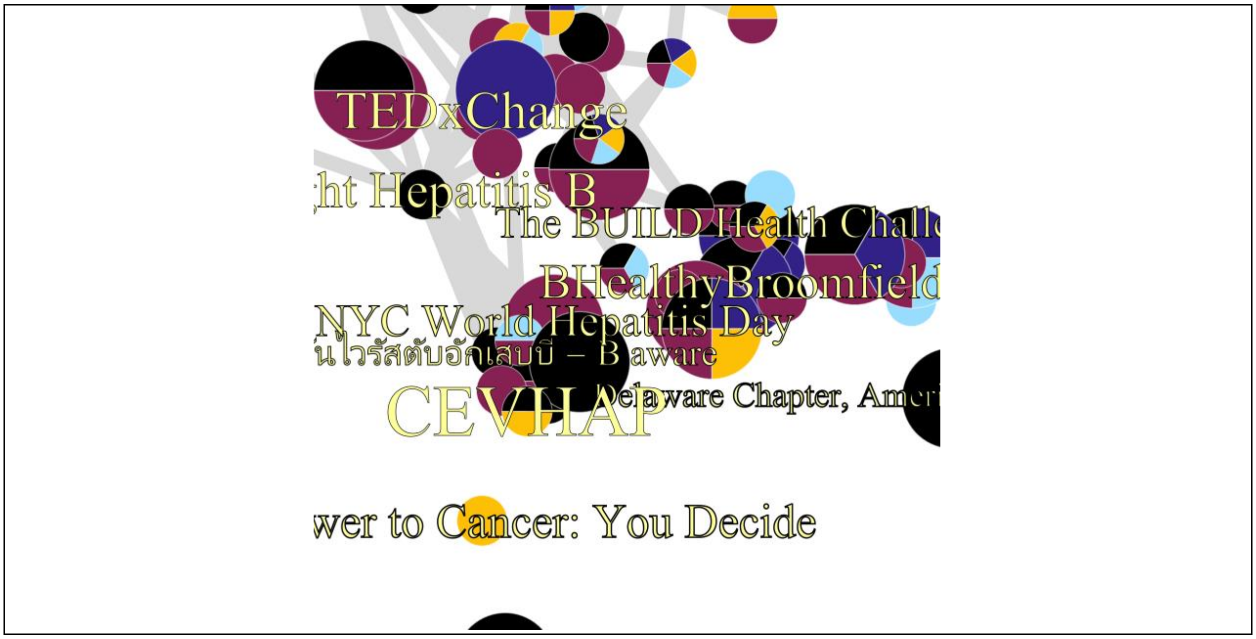

Figure S23: Network zoom-ins for Figs. 1C–G, with node labels.

Section 7: Chi-square test for relationship between topic and geographic glocality

Here we set out to answer the question of whether there is a relationship between the 5 different topics of study and the geographic local/global nature (i.e. geographic glocality) of the communities discussing them. To do this, we employed the chi-square test where:

H0: There is no association between the topics and geographic glocality, i.e., topic is independent of geographic glocality. The phenomenon in the population under statistical test is considered absent;

H1: There is some association between the topics and geographic glocality, i.e., topic is dependent upon geographic glocality.

In this way we can determine if there is a topic--geographic-glocality relationship. In this case, the row variable is geographic glocality, related to whether a community specifies a location in its username, or if the username has a more "global" focus, and column variables are the different combinations of topics, e.g., "COVID-19", "COVID-19 and mpox", etc. The result was statistically significant at the 0.0025 significance level, $\chi 2$ (28, N = 767) = 69.6, p = .0000214. To investigate the degree to which this association is present, we calculated the effect size, Cramér's V, which was .302, and can be considered moderate. Of course, Cramér's V, can be a biased estimator as it increases with the number of cells, thus after applying a bias correction, Cramér's V = .234. This effect size can be considered small, but it is important to remember that oftentimes effects are likely to be small due to the subtlety of the issues - involved—in this case, it's reasonable to assume that there may be a trivial or negligible effect between some of the topic combinations and geographic glocality, and a large effect between others, and .2 is not so small as to be negligible. It is important to keep in mind that all possible combinations of the 5 topics results in 31 different column variables, and the results of the chi-square test might be different had the column variables only been the 5 topics, had all these topics been mutually exclusive categories, with no overlap. As it is, as each observation is not categories to one and only one category, it is not possible to perform the chi-square test to determine if there is an association between only the 5 topics and glocality.

Using the results of the chi-squared test, we can compare the observed values to the expected values: for communities that only discussed 1 topic, for example, 25.21%, 35.14%, 17.65%, 38.71%, and 24.32% of communities discussing COVID-19, mpox, abortion, elections, and climate change, respectively, are local. This is in contrast with the expected percentage, which for all is 31.9%. Thus, while one's expectation that discussions surrounding elections would be highly localized, one might also expect the same of abortion, due to laws, providers, etc. We see however that abortion discussions are instead rather global and deviate the most from the expected value. We also see a difference between values for COVID-19 and mpox, whereas one might have expected them to be closer as this dataset was initially gathered pre-coronavirus pandemic regarding vaccine. One might have further expected COVID-19 discussions to be more localized, due to countries having different isolation, masking, and vaccination requirements, however this is not the case.

<u>Section 8:</u> Use of nVenn diagrams. General system dynamics, glocal breakdown, and comparison to simulation.

As the interactions of dialogues regarding the topics of COVID-19, mpox, abortion, elections, and climate change are complex and can be highly interconnected, untangling them is facilitated through the use of Venn diagrams. However, traditional Venn and Euler diagrams have a few limitations that can make them less attractive for representing complex data. Some of the constraints that impeded the effectiveness of Venn and Euler diagrams for our data are:

1. The inability to show proportional sizes of sets (in this case, "sets" comprise of the posts and pages that discuss the different topics). Traditional Venn and Euler diagrams typically represent sets as circles or ellipses whose areas are proportional to the size of the sets. However, there is a problem with this in that the sizes of the circles or ellipses are not proportional to the sizes of the actual sets being represented. Unfortunately, this means that the diagrams can be misleading when comparing sets of vastly different sizes, especially when the difference in size is not apparent from the diagram.
2. The difficulty in visualizing multiple sets. Traditional Venn and Euler diagrams can be challenging to use when trying to represent more than three sets, since it becomes difficult to visualize the overlapping areas of multiple sets. The diagrams can also become cluttered and hard to read. This is a particular challenge with this dataset, where we are interested in the dynamics of 5 different topics, which overlap in strange, non-uniform manners.

To that end, we therefore turned to the software tool nVennR, which produces generalized, quasi-proportional Venn and Euler diagrams. nVenn diagrams provide a more accurate representation of the size of the sets being compared, as well as more easily allow for the visualization of multiple sets, which is vital for highly complicated datasets such as ours.

How to read these nVenn diagrams is explained in Fig. S20A using a simple example with just 15 communities and 3 topics. By performing regex searches on the posts collected from Facebook communities, these Facebook communities can be categorized according to the topics they post. This is represented on the left of Fig. S20A, where 15 communities have been categorized according to whether they post about any of 3 topics. The communities are labelled with the corresponding topic number, and topics are further distinguished by color. On the right, then, we can observe the intersection, containment, and disjointness of the communities and the topics they are discussing. Furthermore, the area of each region is proportional to the number of communities it contains, and, unlike Venn diagrams, the nVenn diagram does not contain all possible zones, and only depicts relevant relations. We can thus gain an intuitive understanding of the relationship between communities, the topics that interest them, and the overlap there within.

Fig. S20B shows the relations between the 5 topics and all the Facebook communities in the dataset that are actively using them in their discourse. Fig. 1A shows the breakdown of 767 communities which has a population of 81.9 million combined. Community-wise, the regions for discussing only COVID-19 or only climate change have the largest values, at 119 and 113 respectively, however discussing all 5 topics comes in 3rd place, and population-wise has the largest value, at 19.9 million individuals. Comparatively, there are far fewer pages discussing only mpox, abortion, or elections (37, 18, and 31 communities respectively), and there is a lot of crossover chatter particularly within the regions for COVID-19 and climate change particularly among global communities (9.4% of all global communities vs 3.0% of local ones).

We can compare these results to the 1000 simulations we performed, to see how much the data differs. One typical outcome from all those simulations is presented in Fig. S20C, where from quick visual inspection we see that there were no communities discussing all 5 topics, and most communities were predicted to be single- or double-topic focus, e.g., discussing only COVID-19, COVID-19 and mpox, etc. For the simulation, on average only ~2 (±1.14) communities discuss all 5 topics, while ~486 (±15.55) communities are single-topic only—in contrast to the 73 communities engaging with all 5 topics, and only 318 communities which are single-topic focused. From data to simulation, that is a 52.77% increase in single-topic focused communities and 97.05% decrease in 5-topic focused communities. We do see however for both the simulation and the data, that community-wise, the regions discussing only COVID-19 and only climate change are the largest and second largest, though swapped. One might have expectations that as the number of topics a community engages in increases the potential for friction among the members, resulting in primarily 1- or 2-topic focused spaces, which is supported by the simulation, but the reality is the opposite.

We further disaggregated Fig. S20B into global and local communities to investigate how username glocality might influence topic glocality. In addition, we wanted to investigate what, if any, role community vaccine type might play. In Fig. S20B we see that only one topic—the intersection of COVID-19, mpox, elections, and climate change—is dominated by both local and anti-vaccination communities, but investigating local communities only, looking at only those topic intersections with 3% or more of total communities, we see that in Fig. S20D, 8 and 3 regions are dominated by anti-vaccination and neutral communities respectively. In contrast, in Fig. S20E there is a 5-5 split between antis and neutrals. Comparing these diagrams to Fig. S20B, we see despite there being overall less local and less anti-vaccination pages in general in the full dataset (342 local vs 1014 global; 501 anti vs 644 neutral), the general trend in Fig. S20D prevails, with more regions dominated by anti-vaccination communities. If we remove neutral as an option, for Fig. S20B, only 3 regions would be dominated by pro-vaccination communities, none of which contain 3% or more of communities. This was also the case for Fig. S20D, though 12 regions with less than 3% of communities were dominated by pros; for Fig. S20E, only 1 region with over 2% of communities would be dominated by pros, though overall there were 20 regions total once neutral was removed as an option. Thus, while there are pro-vaccine communities getting involved in these discussions, the dialogue is ultimately being dominated and driven by communities who are not actively promoting best-science guidance, and in many cases the communities taking reign are those which actively oppose it, especially at a local level.

To summarize, therefore, Fig. 3 uses these n-Venn diagrams to show the partitioning of topics among communities. These diagrams provide a visualization of topic glocality, and the intersection, containment, and disjointness of communities and their corresponding topics. The area of each region in the diagram is proportional to the number of communities it contains, and it only depicts relevant relations, rather than all possible zones, unlike traditional Venn diagrams. Therefore, nVenn diagrams allow us to intuitively comprehend the relationship between communities, their interests, and the degree of overlap.

We also explored heat plots in conjunction with the nVenn diagrams, in order to visualize better the topic space. Figures 3C,D shows how topics got combined in the communities' discourse during two different time windows: May 12 to May 26 (around the time of the first documented case of the 2022 mpox outbreak in the U.S.A.), and from June 17 to July 1 (around the time the U.S. Supreme Court reversed Roe v. Wade). Its uses a matrix to show the two-topic signal (i.e. a pair of topics). Our analysis, as shown in Fig. 3C–D, reveals that communities were primarily interested in discussing COVID-19 and climate change throughout the period from May 1 to October 17, and that anti-communities dominated discussions regarding diseases and viruses. However, at specific moments during this 6-month period, the

trends varied. For instance, around the first mpox case, "COVID-19 and climate change" was dominated by pros. Additionally, while anti-communities seemed to take charge of the majority of mpox discussions in May, "mpox and abortion" was also dominated by pros. Although "COVID-19 and abortion" was dominated by pros during the two 2-week periods analyzed, this was not the case for the entire study period. Moreover, while "COVID-19 and mpox" was the most prevalent crossover in Fig. 3C, it was short-lived, and "COVID-19 and climate change" dominated in Fig. 3D. Additionally, similar to viral interference, these results suggest that one or two dominant topics could suppress conversations related to other topics. The findings present a positive aspect as they indicate that despite the dominance of anti-communities and neutrals in the conversations across different topics, pros can sometimes take charge of the discussion. As there have been several breakthrough cases on a shorter timescale, it may be possible to enhance the impact of pros in the system.

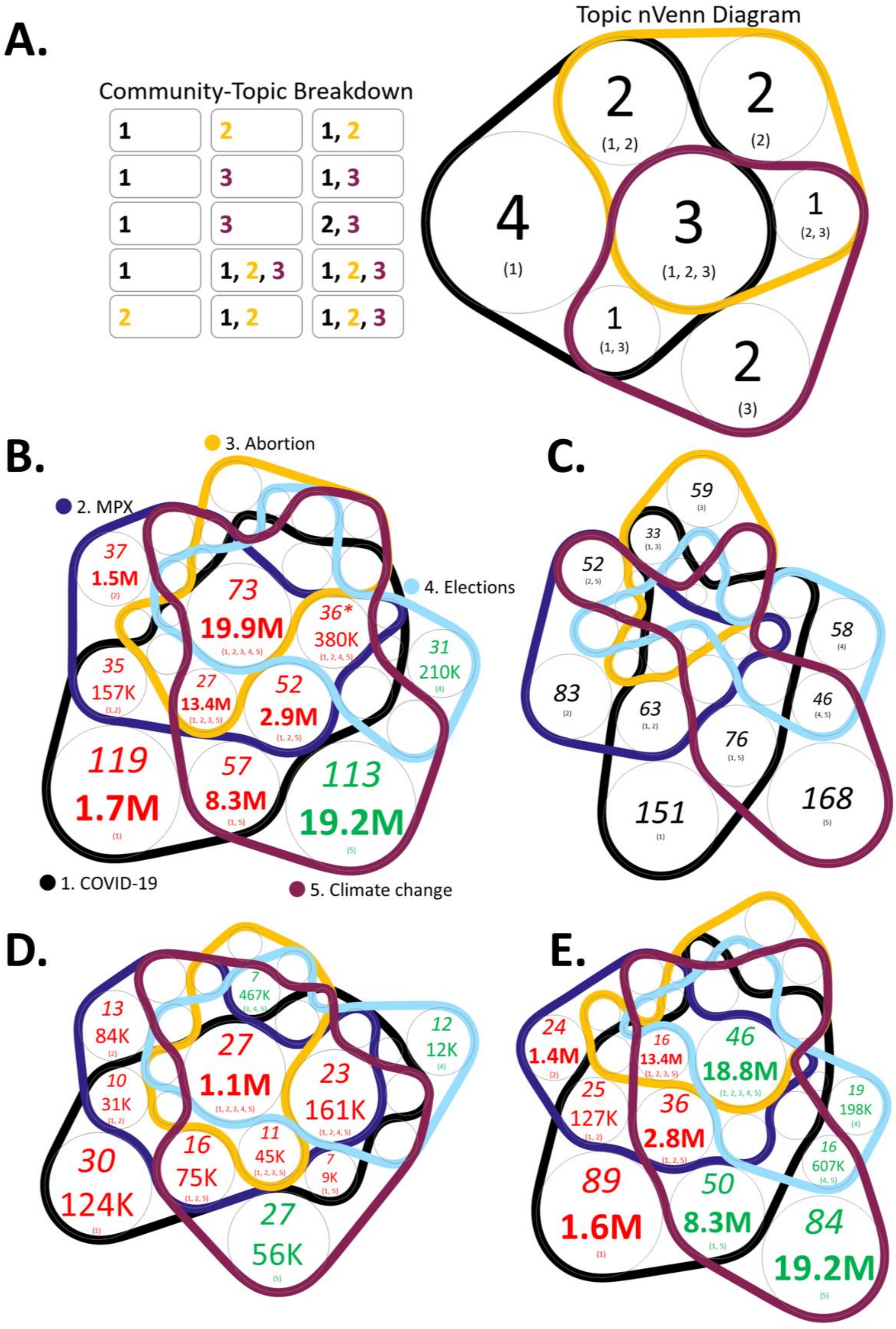
A.
Community-Topic Breakdown
1
2
1, 2
1
3
1, 3
1
3
2, 3
1
1, 2, 3
1, 2, 3
2
1, 2
1, 2, 3
Topic nVenn Diagram
2
(1, 2)
2
(2)
4
(1)
3
(1, 2, 3)
1
(2, 3)
1
(1, 3)
2
(3)
B.
3. Abortion
2. MPX
4. Elections
1. COVID-19
5. Climate change
37
1.5M
73
19.9M
36*
380K
31
210K
35
157K
27
13.4M
52
2.9M
119
1.7M
57
8.3M
113
19.2M
C.
59
33
52
58
83
63
46
76
151
168
D.
7
467K
13
84K
12
12K
27
1.1M
23
161K
10
31K
11
45K
16
75K
7
9K
30
124K
27
56K
E.
24
1.4M
16
13.4M
46
18.8M
25
127K
36
2.8M
19
198K
16
607K
89
1.6M
50
8.3M
84
19.2M

Figure S24: Comparison of topics' discussion across Facebook communities (COVID-19, black; mpox, dark purple; abortion, gold; elections, light blue; climate change, dark magenta). In the circles, only regions that contain 3% or more of communities include text. (A) Schematic illustrating 15 communities (left panel, each rectangular box is a community) with the topics that each discusses (each topic is a colored number in each box). This partitioning is represented by the nVenn algorithm in the right panel. There are 4 communities, for example, that posted about Topic 1 only, whereas 3 communities posted about Topics 1, 2, and 3. The corresponding intersections in the nVenn diagram are sized proportionately. In (B)-(C), italics numbers represent the number of communities whereas the bottom number represents their total population; total populations of a million individuals and larger are bolded; red text indicates that anti-vaccine communities were the most prevalent, whereas green text indicates neutral communities were. (B) Breakdown of all FB communities involved in discussing one or more of the topics. An asterisk next to the number of communities means that there are more local than global communities. (C) Result from a single representative simulation, demonstrating that just based on the vaccine-categorization of FB communities, one would expect very few communities to be discussing more than 2 topics at a time. Indeed, most communities are expected to be single or double topic focused at most. In this simulation, no pages are discussing all five topics, in contrast to the actual data. (D) Breakdown of all local communities. (E) Breakdown of all global communities.

Section 9:

Relationship between the number of topics and geographical glocality.

To explore the correlation between vaccine category, geographic glocality, and topic glocality, we also employ another approach: to categorize topic glocality into various subsets, i.e., we explore the various subsets of topics that exist, from having just one topic to all five. There are five different subsets for one element, ten for two, ten for three, five for four, and one for five. For instance, there is only one way to talk about all five topics, but five ways to discuss only one of the five topics. We started by determining the number of communities discussing 1 to 5 topics, and then examined the vaccine and geographic breakdown of these communities. Once we identified the communities and their topic discussions, we could then tally the number of individuals across communities, allowing us to understand the distribution of individuals in this system. However, it is worth noting that the dynamics of communities versus individuals can be vastly different, as some communities have tens of members while others have millions, and this is reflected in the data presented in Figure 2. In particular, Fig. 2B, which focuses on the number of communities, shows a general "U" shape with regards to geographic glocality and vaccine category breakdowns. Interestingly, we found that local anti-communities were proportionally more likely to discuss all five topics, which may suggest a link between geographic locality and anti-vaccination sentiment. However, when examining Fig. 2A, we observe a complete reversal of this trend. This is because in our dataset, neutral communities are extremely popular, with millions of followers. Thus, although local anti-communities may be actively involved in all 5 topics, the individuals in global and local neutral communities are heavily embedded in 5-topic discussions. In addition, we notice that the overwhelming majority of individuals across communities are involved in 5-topic discussions, unlike the community breakdown shown in Fig. 2B. Furthermore, while there are few individuals in global pro-communities contributing to 5-topic discussions, the same cannot be said for local pro-communities.

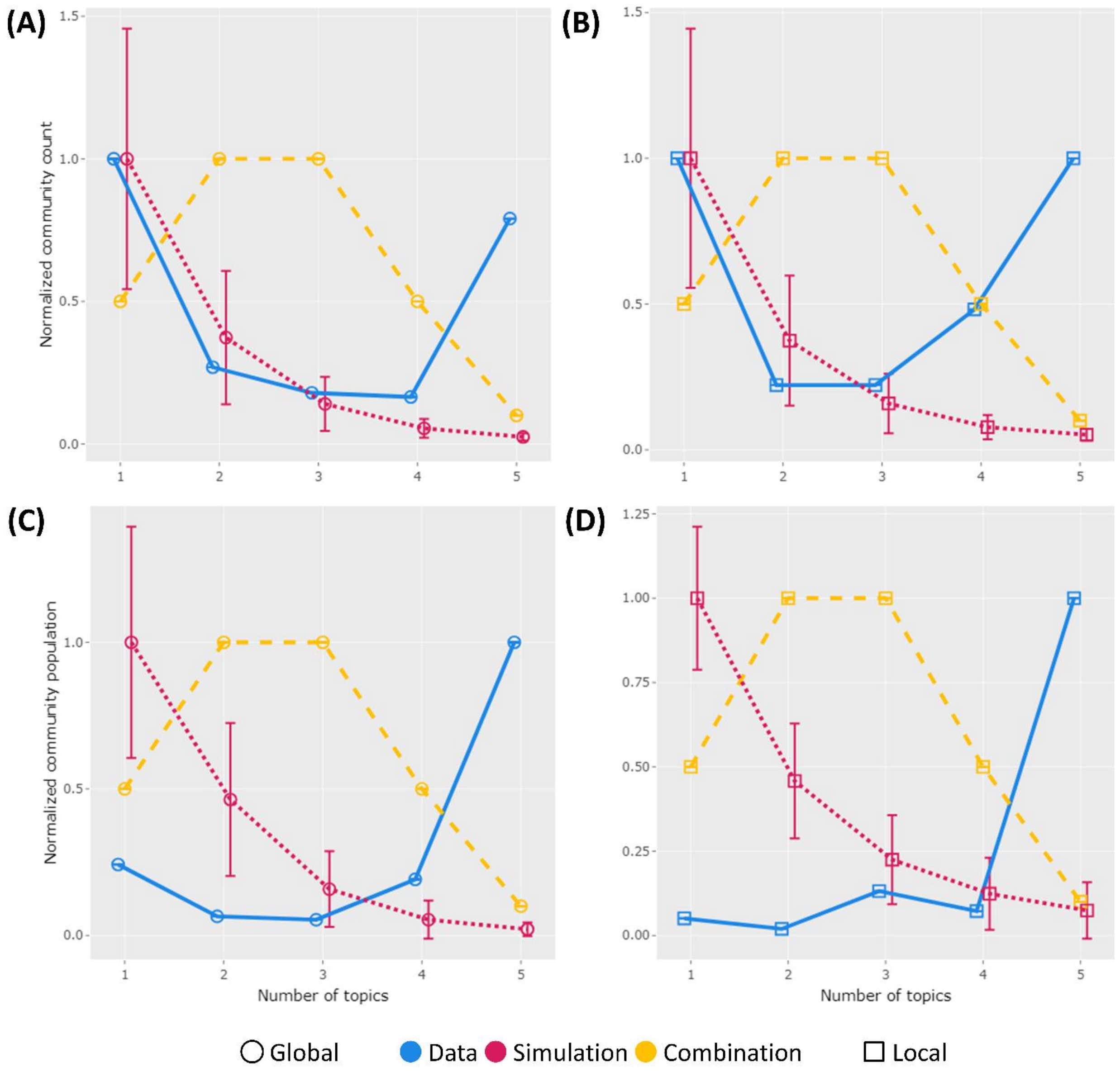

Figure S25. Glocality of communities and populations compared to estimates and simulation. Blue solid line for data, magenta dotted line for simulation, and yellow dashed line for estimate. Plots on left are for global data whereas the right are for local data. Simulation is based on the idea that the number of communities discussing x topics is based on the vaccine type of the communities, and has a decreasing, concave up shape in every case. Estimate is based on the idea that the number of communities discussing x number of topics is based on combinations. For example, for communities discussing only one topic out of 5 possible topics, we would expect C(5,1) communities posting about only one topic, or 0.5 when normalized through division by maximum. (A) Comparison of global communities (solid blue) discussing x number of topics (ranging from only 1 to all 5) to simulated (dotted magenta) and estimated (dashed yellow) values. The actual data curve has a "U" shape and the simulation deviates from data at 4 topics. (B) Comparison of local communities (solid blue) discussing x number of topics (ranging from only 1 to all 5) to simulated (dotted magenta) and estimated (dashed yellow) values. The actual data curve has a "U" shape and the simulation deviates from data at 4 topics. In contrast to the global data, the estimate only predicts the

normalized number of communities for 4 topics. (C) Comparison of global populations (solid blue) discussing x number of topics (ranging from only 1 to all 5) to simulated (dotted magenta) and estimated (dashed yellow) values. The "U" shape has a lower left end compared to the right end for the actual data curve and the simulation predicts only the normalized population value for 3 topics. (D) Comparison of local populations (solid blue) discussing x number of topics (ranging from only 1 to all 5) to simulated (dotted magenta) and estimated (dashed yellow) values. The actual data curve has an increasing, concave up shape and how simulation predicts only the normalized population value at 3 and 4 topics.

One might have imagined that as a community tackles more topics, the number of potential flashpoints for internal disagreements increase, which in turn would result in fewer communities engaging in a greater number of topics. Hence the curves in Fig. 2 would all be expected to decay with increasing number of topics -- yet the opposite happens in Fig. 2. This may be because social media's ability to connect like-minded individuals who share similar beliefs, isolates them from those with differing viewpoints (66) hence leading to a general "distrust" that transcends topics. This can result in an increasing number of communities engaging in an increasing number of topics.

Using combinatorics, we might explore if Fig. 2 can be explained mathematically by saying that there are $C(5, n)$ ways to engage with n topics, where $C(5, n)$ is the number of ways to choose a sample n of topics from the 5 topics in this study. Hence, out of 31 communities, one might expect there to be $\frac{5}{31}.31 = 5$ who worry about 1 topic, 10 who worry about 2, 10 who worry about 3, 5 who worry about 4, and 1 who worries about 5. Since our dataset is larger than 31 communities, these values can be scaled accordingly. In order to compare the expectations of our simulation and combinatorics with the actual data, we can refer to Fig. S21, which shows the data (solid blue) broken down into global and local communities (top row) and their populations (bottom row) on the left and right side, respectively. The simulation results are represented by a dotted magenta line, while the dashed yellow line represents our expectations based on combinatorics. To make the datasets comparable, all of them have been normalized by their maximum value. The simulation assumes that communities discussing topics are a random sample based on their vaccine categories, with a community's vaccination status being the primary factor in determining its topic engagement. However, as shown in Fig. S21, none of these expectations match the actual data except in specific circumstances.

In Figs. S21A–B, the simulation predicts engagement in 1, 2, and 3 topics, but deviates greatly for 4 and 5 topics. The only case where the simulation matches the data is for 4 topics in Fig. S21B. Differences between global and local trends for both communities and community populations are subtle, but for Fig. S21A vs Fig. S21B, 4 topics deviate more quickly and dramatically for local communities. Proportionately, more local communities discuss 5 topics than global. Both data curves have a "U" shape. In Fig. S21C, the simulation only predicts 3 topics, while in Fig. S21D, it predicts 3 and 4 topics. Global and local trends in community populations differ, with Fig. S21C having a "U" shape and Fig. S21D having an almost increasing, concave up shape. The largest populations engage in all 5 topics. These "U" shapes in Figs. S21 might be explained by our expectations: engagement in up to 3 topics decreases with increasing potential for disagreement, but after this point, the number of communities involved sharply increases. Hence, we are left with the following as a candidate explanation: it is because social media's ability to connect like-minded individuals who share similar beliefs, isolates them from those with differing viewpoints (66) hence leading to a general "distrust" that transcends topics. This can result in an increasing number of communities engaging in an increasing number of topics.

Topic heatmaps:

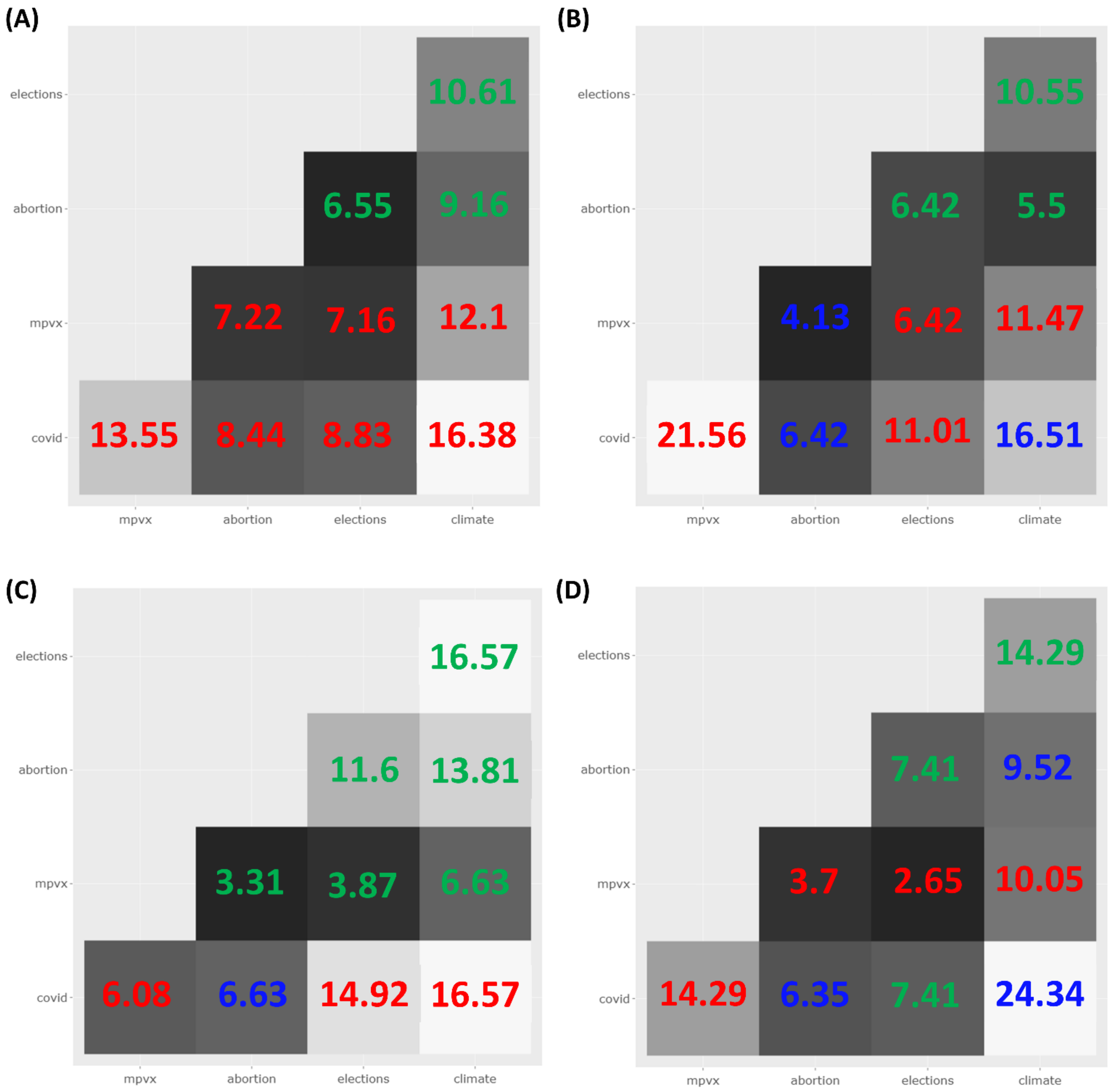

Figure S26: Heatmaps showing prevalence of cross-topic discussions. Symmetric squares are left empty since "Topic A and Topic B" is equivalent to "Topic B and Topic A". Colored tiles indicate magnitude of communities discussing the paired topics, with black being the lowest and white the highest. Text color denotes the dominant vaccine stance within those discussions. (A) 1 May 2022 - 17 October 2022: Most common were COVID-19-climate change, COVID-19-mpox, and mpox-climate change. No cross-topics were pro-dominated. (B) 12-26 May around first U.S. mpox case on 19 May: Highest interest in COVID-19-mpox and COVID-19-climate change. Unlike overall trends, some COVID-19 pairings were not anti-dominated, while abortion was split between pro/neutral stances. (C) 17 June - 1 July around Roe v. Wade reversal on 24 June: Spikes in abortion-elections and abortion-climate change. Climate change pairings dominated. Only COVID-19-abortion was pro-dominated. (D) 3-17 October before student loan forgiveness rollout: Continued interest in

COVID-19-climate change and COVID-19-mpox. COVID-19-abortion and elections-climate change also pro-dominated in this period.

To examine how communities discussed combinations of topics, we generated heatmaps at three key time points: (1) 12-26 May around the first U.S. mpox case; (2) 17 June-1 July around the reversal of Roe v. Wade; and (3) 3-17 October before U.S. student loan forgiveness applications opened, a salient election issue. Figure S__ shows that over the full study period (1 May - 17 October), COVID-19 and climate change interactions were common, with anti-communities prevailing in disease/virus conversations. However, narrowed snapshots revealed fluctuations. Around the first mpox case, more pro-communities discussed "COVID-19 and climate change". In early October as well, this pairing was pro-dominated. While anti-communities led most mpox discussions in May, "mpox and abortion" tilts toward pro-communities. "COVID-19 and abortion" also skewed pro during the 2-week windows, unlike the overall 6-month trend. Thus, while anti/neutral communities generally dominated cross-topic discourse, pros occasionally controlled specific conversations over short timescales. Leveraging these breakthroughs may increase pro influence on the broader system.

Section 10: Details of the geographic scale simulation

We also developed a simulation to test the relationship between the local and global communities in the dataset. We are interested in investigating this relationship because, by pure numbers, every one of the 5 topics has less global communities/more local communities involved than compared to the full dataset, that is to say, local communities are overrepresented in every topic we investigated. Using the two proportions test, with the alternative hypothesis that the proportion of global pages is less compared to the full dataset, all topics were statistically significant at the 0.27% level, except for "abortion". The broad trend is thus that local communities are overrepresented in topics compared to the full dataset, and more important to this system than one may think if they just take into consideration all communities involved in vaccine discourse.

Hence, we designed a simulation (used to generate Fig. 4 of the main paper) to explore the glocal cohesiveness of the network, to see if official messaging targeted at the different organizational scales (local, regional, national) can make an impact, and if that impact is sustainable. The simulation works in the following manner: each time step, a local node from the list of active local nodes is randomly selected to be deactivated. The network is then updated to remove all links to/from this node, and the number of nodes still in the giant component is recalculated. Then, at every time step, all deactivated local nodes have a chance to reactivate based on the proportion of global nodes it follows as compared to the total number of nodes it follows. This means that if a local node follows no global nodes, i.e., a local community only follows other local communities, that node will have no chance of reactivating because the probability is 0%. The simulation was performed 1,500 times, and plotted results are the averages of that.

In this dataset, there are 106 such nodes out of 185 local nodes total that posted concerning at least one of the 5 topics. There is no node that follows exclusively global nodes; thus, the highest proportion is 88.89%. The range of the chance of reactivation, excluding 0%, are: 88.89% to 10% (when the proportion is divided by 1), and 1.778% to 0.2% (when the proportion is divided by 50).

One might think that even with such high chances of reactivation, it would be possible to deactivate all local nodes in the giant component. However, we see from Fig. 4 that this is simply not the case. In fact, the proportions had to be divided by 50 before we were able to enter a regime where it was possible to deactivate all local nodes in the GCC. This means that the highest proportion was 1.778%—in other words, even there is even a 2% chance for even one local node to reactivate after being deactivated with official messaging targeted at the local level, it is not possible to remove all local nodes from the GCC. Additionally, even in this case the average hovers above the minimum value possible for the GCC with no local nodes activated, because even with such low probabilities, in general it is not completely possible to deactivate all local nodes all the time or at once.

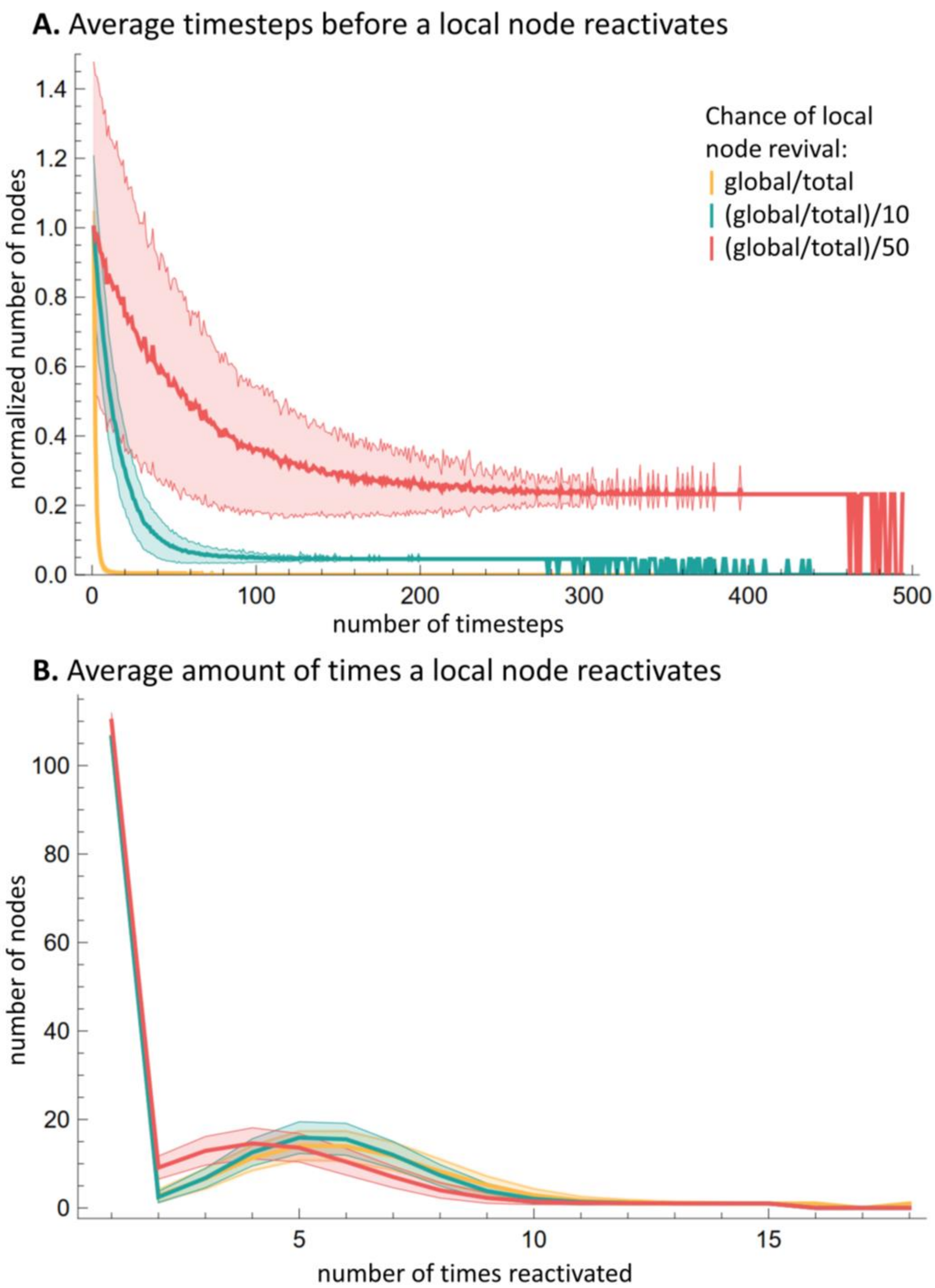

Figure S27: Results from the glocality simulation. (A) Counts normalized to facilitate comparison between the 3 curves. These curves depict the average amount of time a node is deactivated, with 1σ interval bands. As one might expect, nodes spend the least amount of time deactivated when the chance of reactivation is higher. Curves become choppy at larger numbers of timesteps because comparatively fewer nodes remain deactivated for that long, meaning there are fewer values over which to compute the average. (B) These curves depict the average amount of times a node is reactivated, with 1σ interval bands. As one might expect, nodes are more frequently reactivated when the chance of reactivation is higher, though it is not a major increase.

The average times deactivated before reactivation are (1.30091, 18.8005, 66.6677) and the average times reactivated are (2.12106, 2.0157, 1.53282) for the curves with the proportion of activation divided by 1, by 10, and by 50, respectively. For Fig. 4, the only curve for which we can calculate an average number of time steps until all local nodes are deactivated is the one where the proportion is divided by 50. That value is 492.131 ± 14.3054 steps. Additionally for Fig. 4, the maximum steps until half of all local nodes are deactivated was (374, 230, 137) while the minimum was (177, 135, 100) for the curves with the proportion of activation divided by 1, by 10, and by 50, respectively.

For the results in the main body of the paper, the averaged results of these simulations are depicted in Fig. 4A, where curves are the mean plus-minus one sigma. It was found that it was not possible to remove all local nodes from the giant component even with official messaging targeted at the local level, and even with low probabilities, it is not completely possible to deactivate all local nodes all the time or at once. The only curve for which the average number of time steps until all local nodes are deactivated could be calculated was the one where that proportion of global to all fanned pages is divided by 50, which can be seen in Fig. 4A. For that simulation, the highest chance of reactivation was 1.778%, and the smallest, non-zero chance was 0.2%%—in other words, if there is more than even a 2% chance for one local node to reactivate after being deactivated with official messaging targeted at the local level, it is not possible to remove all local nodes from the giant component. The average times deactivated before reactivation are (1.30091, 18.8005, 66.6677) and the average times reactivated are (2.12106, 2.0157, 1.53282) for the curves with the proportion of activation divided by 1, by 10, and by 50, respectively. For Fig. 4A, the only curve for which we can calculate an average number of time steps until all local nodes are deactivated is the one where the proportion is divided by 50, and that value is 492.131 ± 14.3054 steps. ***The simulation results reveal the remarkable interconnectedness of this network across geographic glocal scales, making it resistant to messaging targeted at only one scale. To reach these communities, official messaging cannot be exclusively directed at the local level.***

<u>Section 11:</u> Details of the topic glocality simulation

We then developed a simulation to evaluate the efficiency of de-bunking or pre-bunking in this system and to assess the impact of both single and multi-topic messaging in achieving that objective. For this simulation, topics are chosen at the most granular or "local" level, that is to say, a "topic", can be "COVID-19", "COVID-19 and mpox", "COVID-19, mpox, and climate change", etc. This simulation assumes that the de- or pre-bunking messaging aimed at this topic is the "ultimate" messaging possible, that it would tackle any concern someone might have on that topic. This means if the topic is "COVID-19", debunking messaging is only aimed at "COVID-19"; if the topic is "COVID-19 and mpox", messaging is aimed at "COVID-19 and mpox".

To that end, the simulation works in the following manner: at each time step, a node/Facebook page is randomly chosen. If that page was posting about that topic, it will no longer do so. For example, if the topic is "COVID-19 and mpox", and the randomly chosen node was posting about "COVID-19", "mpox", or "COVID-19 and mpox", it will stop doing so. We keep track of all nodes that have been randomly chosen and were posting about the topic. As with the geographic glocality simulation, it is possible for nodes to revert back to posting about this topic. Regardless of reversion method, the probability of reversion is based on the proportion of followed pages discussing the topic to all followed pages. For example, if a page follows 5 other pages, the messaging is only targeted towards COVID-19, and out of those 5 pages only 2 posts about COVID-19, then that page has a 2/5 chance of reverting back to posting about COVID-19.

There were several ways in how reversion could be handled, from most permissive to least permissive. The least permissive revision method is only those nodes that were posting about the topic could revert to posting about it. The most permissive method is that any node, including those that were not originally posting about the topic, could end up returning to posting about the topic (in the case of nodes that weren't originally discussing it, they are simply picking up the topic for the first time). The results of the simulation in Fig. 4B is for the simulation that uses the least permissive method, however we did run the simulation 500 times using the most permissive method for comparison.

The results in Fig. 4B were generated as follows: the curve for one topic is the average of the results for messaging that only focused on "COVID-19", "mpox", "abortion", "elections", or "climate change". The other curves are also averages, meaning for the curve for two topics, it is the average of the results for messaging focused on "COVID-19 and mpox", "COVID-19 and abortion", "mpox and elections", etc. The curves in Fig. 4B separated out are:

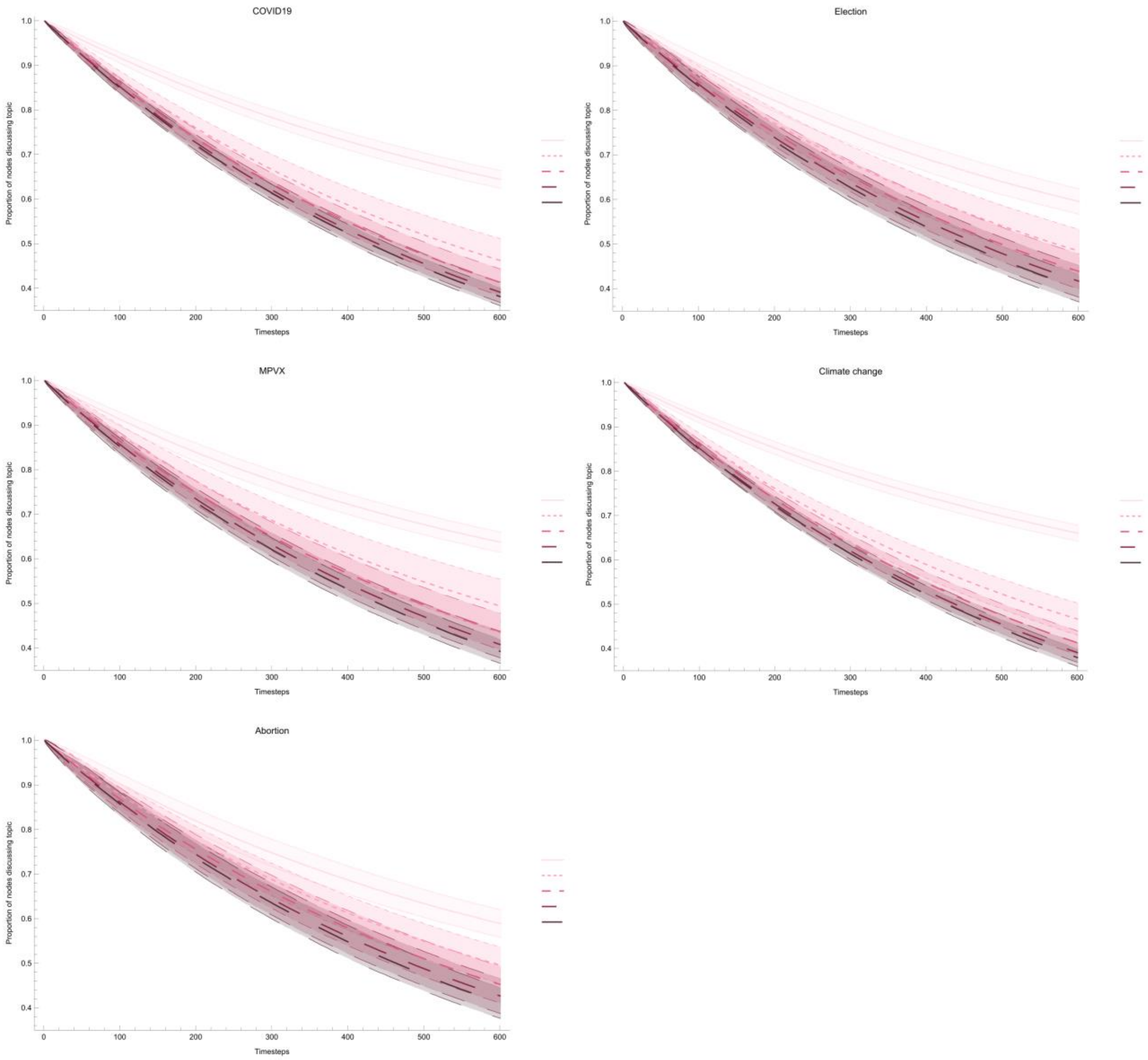

Figure S28: Curves from Fig. 4B broken into individual topics. The general trend seen in Fig. 4B is present for all topics, though sometimes the difference between single and two-topic messaging is more (climate change) or less (abortion) extreme.

For the most permissive revision method, the compliment to Fig. 4B is:

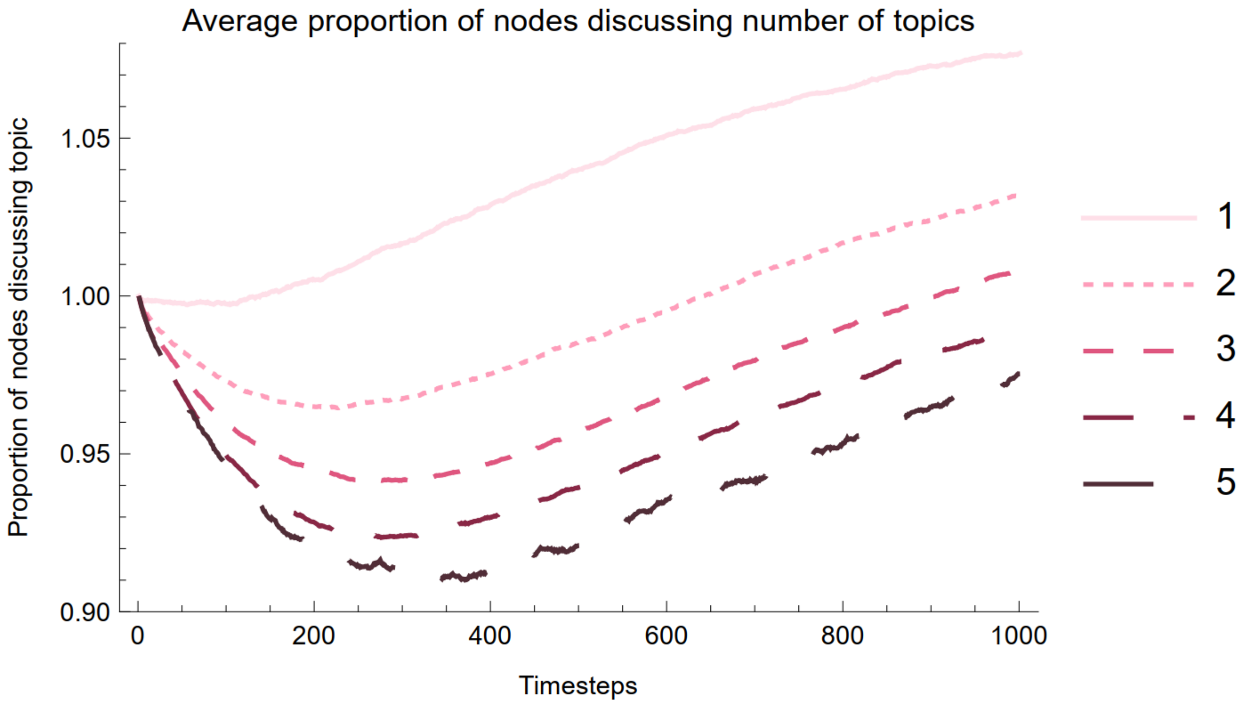

Figure S29: Topic glocality simulation using the most permissive reversion method, where any node can pick up the topic used in the debunking messaging.

We can see this plot has some behavior not present in Fig. 4B. To about 300 steps, all curves excluding the single topic curve trends downwards, until they start trending upwards. This is because these curves are the result of averaging all the curves for the different topics. To get a better sense of what is going on, we separate out the averaged curves into their individual topic curves:

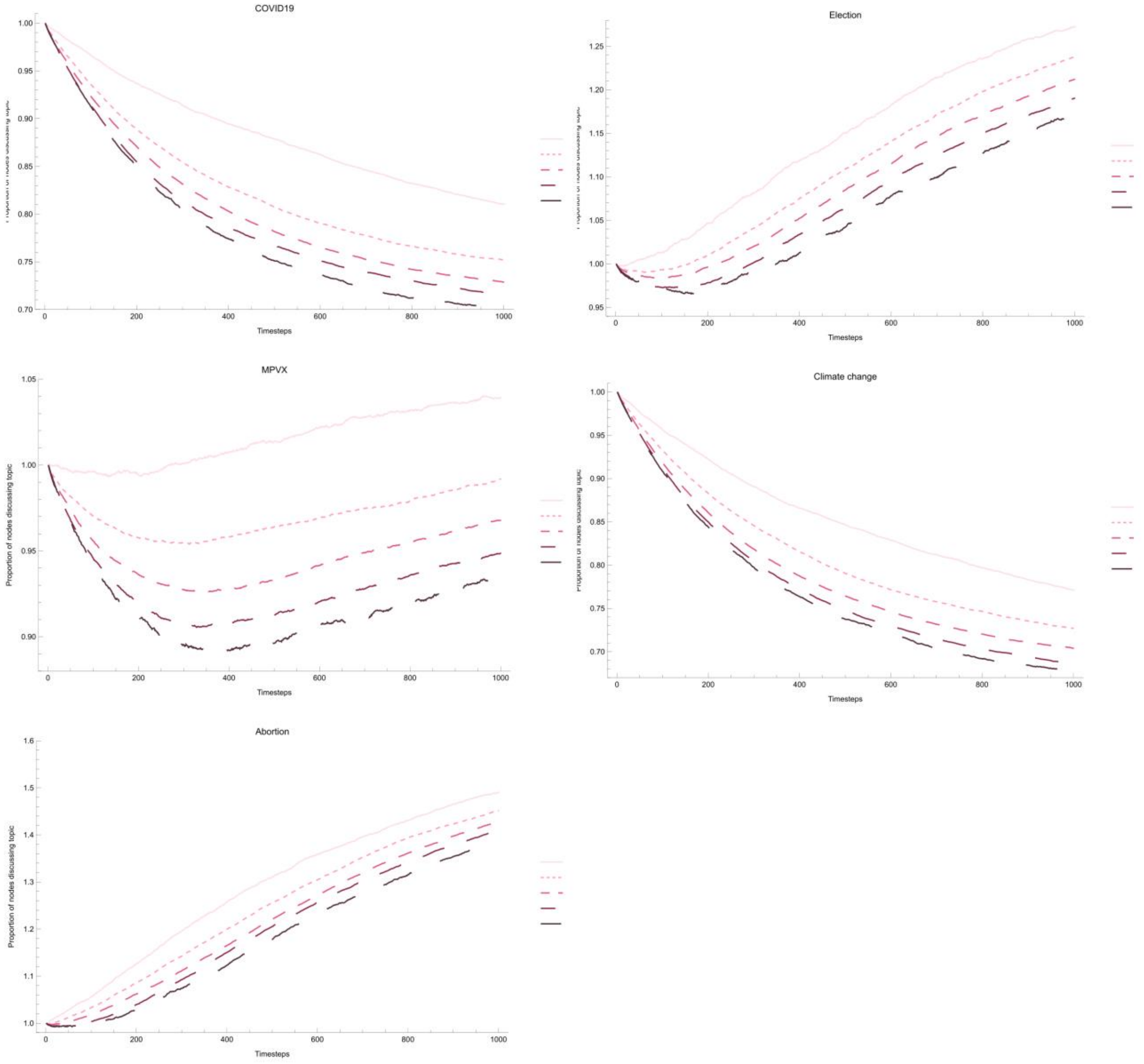

Figure S30: Curves from Fig. S24 broken into individual topics. While for COVID-19 and climate change, there is a general downward trend, for abortion and election, there is a general upward trend. mpox initially trends downwards then upwards, likely due to the simulation hitting a critical point where the reversion method overwhelms the topic debunking method.

Here we see that for COVID-19 and climate change, there is a general downward trend. Abortion and election, however, have upward trends, and how quickly those curves increase is impacted by the messaging. These upward trends are because while the smallest number of pages are discussing abortion and elections, pages that discuss these topics are well-embedded in the system, meaning that the chance of picking up these topics is high. mpox is the odd one out and is similar to the general trend seen in Fig. S24. The way this simulation works is that every node that has been randomly selected henceforth has the chance of reverting back to posting about the topic every step. That means every step of the simulation, there are more and more pages that can potentially pick the topic back up. For mpox, the simulation hits a critical point where the debunking messaging is running up against the interconnected nature of the nodes

discussing mpox (notice that the lower end of the plot is only 0.9, thus 90% of nodes originally posting about mpox are still posting about mpox). The debunking can't keep up with the reversion, and thus the curves start increasing. Thus, for the averaged curves in Fig. S24 we see the impacts of the abortion, election, and mpox curves against the COVID-19 and climate change curves, and since the number of pages that cease discussing a topic grows slower than the amount of pages that can pick up a topic, after about 300 steps we see the averaged curves start increasing. Importantly in all plots however we see that increased topic messaging has an impact on this growth. In the cases of COVID-19 and climate change, increased topic messaging increases how many pages drop a topic, whereas for abortion, elections and mpox, it decreases how many pages pick up a topic.

While these simulations assume that pages adopt topics based solely on what their followers are discussing, we recognize that this oversimplifies how people decide what to post about. Nonetheless, the model provides a reasonable, simplified approximation of the dynamics, even if it may not be a perfect or completely accurate representation.

Therefore, to summarize, the simulation results in Fig. 4B show the average and one standard deviation of 1,000 runs and display a general downward trend for both single and multi-topic messaging, indicating that single-topic official messaging has some effect in terms of reducing discussion on that topic, though the simulation results are beginning to plateau, indicating there is a upper limit to single-topic messaging. However, for two-topic messaging, the proportion of nodes discussing the topic is less than 50% in less than 600 steps, and it decreases faster with more topics. The results for 3-, 4-, and 5-topic messaging are similar, suggesting that while it is worthwhile to craft targeted 2-topic messaging, there is little to gain from expanding it to 3 or more topics. Heat plots from Fig. 3C–D reveal that some topics, such as COVID-19 and mpox or COVID-19 and climate change, are highly correlated, making it worth the effort to create targeted, official messaging that focuses on debunking misconceptions about the two. While 5-topic messaging may provide the best results, it is challenging to imagine a debunking message that targets COVID-19, mpox, abortion, elections, and climate change, and the simulation shows that there would be minimal reason to do so, as the improvement from 2 to 5-topic messaging is not nearly as great as from 1 to 2-topic messaging. as the results from 2-topic messaging are satisfactory. Therefore, it would be worth the effort to craft targeted official messaging that focuses on debunking misconceptions about highly correlated topics, and it is not necessary to expand that messaging to cover a large number of topics, as the benefits of doing so are minimal. Overall, these results suggest that targeted, multi-topic messaging can be a useful tool in reducing (dis)trust on social media.
